# Sensing in Soft Robotics

**DOI:** 10.1021/acsnano.3c04089

**Published:** 2023-08-02

**Authors:** Chidanand Hegde, Jiangtao Su, Joel Ming Rui Tan, Ke He, Xiaodong Chen, Shlomo Magdassi

**Affiliations:** † School of Materials Science and Engineering, Nanyang Technological University, Singapore 639798, Singapore; ‡ Singapore-HUJ alliance for Research and Enterprise (SHARE), Campus for Research Excellence and Technological Enterprise (CREATE) Singapore 138602, Singapore; § Casali Center for Applied Chemistry, Institute of Chemistry, The Hebrew University of Jerusalem, Jerusalem 91904, Israel

**Keywords:** soft robots, actuation mechanisms, materials for soft robots, flexible/stretchable sensors, multimodal sensing, signal processing, soft robotic control, prosthetics, exosuit, industry leaders in soft grippers

## Abstract

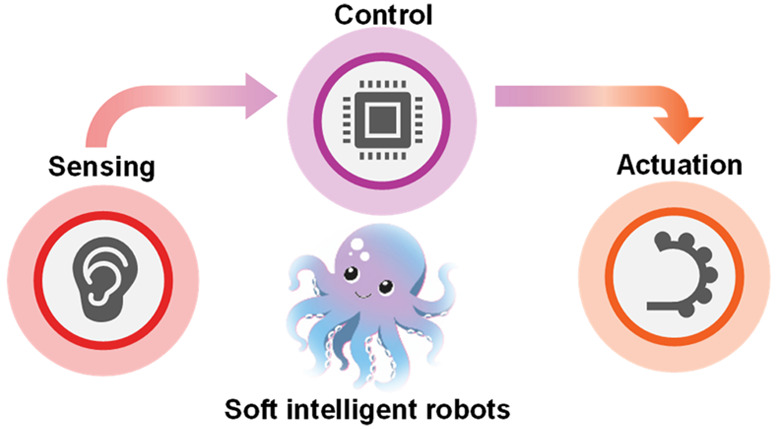

Soft robotics is an exciting field of science and technology that enables robots to manipulate objects with human-like dexterity. Soft robots can handle delicate objects with care, access remote areas, and offer realistic feedback on their handling performance. However, increased dexterity and mechanical compliance of soft robots come with the need for accurate control of the position and shape of these robots. Therefore, soft robots must be equipped with sensors for better perception of their surroundings, location, force, temperature, shape, and other stimuli for effective usage. This review highlights recent progress in sensing feedback technologies for soft robotic applications. It begins with an introduction to actuation technologies and material selection in soft robotics, followed by an in-depth exploration of various types of sensors, their integration methods, and the benefits of multimodal sensing, signal processing, and control strategies. A short description of current market leaders in soft robotics is also included in the review to illustrate the growing demands of this technology. By examining the latest advancements in sensing feedback technologies for soft robots, this review aims to highlight the potential of soft robotics and inspire innovation in the field.

## Automation and Industry 4.0 Needs

Rapid industrialization to meet the necessities of human needs has resulted in the widespread adoption of automation in manufacturing, packaging, medicine, agriculture, and the food industry. Robots equipped with sensors have enabled industry 4.0 technologies, such as the Internet of Things (IoT), big data analytics, and artificial intelligence for monitoring and controlling the function of these robots. Currently, rigid robots are widely adopted to be highly reliable, accurate, and durable. However, handling more delicate and fragile objects requires robots with soft interfaces, which has resulted in an increased interest in soft robotics. As the name suggests, soft robots are made of soft materials or have a soft interface covering a rigid skeleton/support. Soft robots are flexible and quickly adapt to the shape of the object. Mechanical compliance significantly reduces the applied pressure for grasping the objects, unlike rigid robots whose contact area is limited due to the rigid form of the grippers. Soft robots are also highly suited for applications such as healthcare where safety is a priority. Also, owing to their soft and jointless body, soft robots can access remote areas and perform functionalities^1^ that are difficult to accomplish using hard robots. Soft robots can come in various shapes, forms, and actuation mechanisms based on the type of application, such as gripping, locomotion, underwater exploration, flight, and so on. The primary focus of this review is on sensors integrated into soft robotic grippers, which are gaining increased interest in agricultural harvesting, warehouse management, and health care.

## Challenges in Soft Robotics and the Need for Sensing

Soft robots possess distinct characteristics such as large degrees of freedom, high mechanical compliance, and the ability to undergo deformation^2^ through both internal drive and external loads.^3^ This makes it challenging to detect the shape and location of each part of the robotic gripper accurately in three-dimensional (3D) space. Unlike rigid robots that rely on accurate control of joints and limbs, soft robot control requires morphological computation,^4^ which depends on the robot’s morphology and material properties. This necessitates the use of soft materials with programmable material properties. However, modeling the dynamics of soft materials is much more complicated^5^ compared to the simple kinematics of rigid joints, making it challenging to control and monitor the shape and position of different parts of the soft robot.

To overcome this challenge, integrating sensors into soft robots is crucial. These sensors enable the monitoring and control of the shape and position of different parts of the soft robot. Additionally, the sensors can enhance a soft robot’s awareness of external stimuli such as temperature,^6^ pH,^7^ chemicals,^8^ pressure,^9^ light,^10^ and sound,^11^ which significantly widens the scope of the application of soft robots. With the help of sensors, soft robots can perform complex tasks in diverse fields such as healthcare^12,13^ agriculture,^14,15^ and warehouse management, among others. Integrating sensors into soft robots poses a challenge since the sensors must be capable of stretching, bending, and deforming along with the robot without hindering the free movement of the robot while preserving its softness during sensing. This results in nonlinearities, singular configurations, and nonunique mappings associated with soft sensors.^16^ Addressing these issues requires sophisticated modeling and analysis of the sensor data to accurately map environmental stimuli to the sensor data. Moreover, to increase the functionality of the sensors, soft robots must be equipped with high spatiotemporal resolution sensors.^2^ However, this generates large volumes of data that must be processed rapidly^17^ for closed-loop monitoring and control. With the growing demand for soft grippers, there is an increasing need for the integration of various sensors, such as fruit ripeness, temperature, proximity, food spoilage, pH, gas sensors, and many more. Therefore, selecting the appropriate sensing mechanism, the number of sensors, and their intelligent integration are crucial to minimize the computational load^18^ on the microcontroller, realizing efficient sensor integration. With the successful integration of sensors into soft robots, they can perform complex tasks in various applications with enhanced accuracy and efficiency.

To achieve this, appropriate changes in the design and choice of manufacturing process are necessary. One emerging field that enables the integration of multimaterials into complex shapes is multimaterial additive manufacturing. This technique can aid in the efficient and reliable fabrication of soft robots with integrated sensors. Moreover, minimizing the number of steps involved in the fabrication of the smart soft robot and automating the process are essential to increasing the reliability and repeatability of both actuation and sensing functionalities.

In this review, we focus on the types of sensors used for soft robots and briefly discuss various components of a smart soft robot to showcase the scope of opportunities for innovation. We also explore various sensing technologies researched by the community over the past decade for integration into soft robots. Furthermore, we examine the actuation mechanisms and typical materials used in soft robots and deliberate on potential use cases of intelligent soft robots. Finally, we list some of the current industry leaders in the field of soft robotic grippers and argue for the need for appropriate methods for manufacturing smart soft robots to improve their functionality.

## Key Components of a Smart Soft Robot

**Figure 1:**
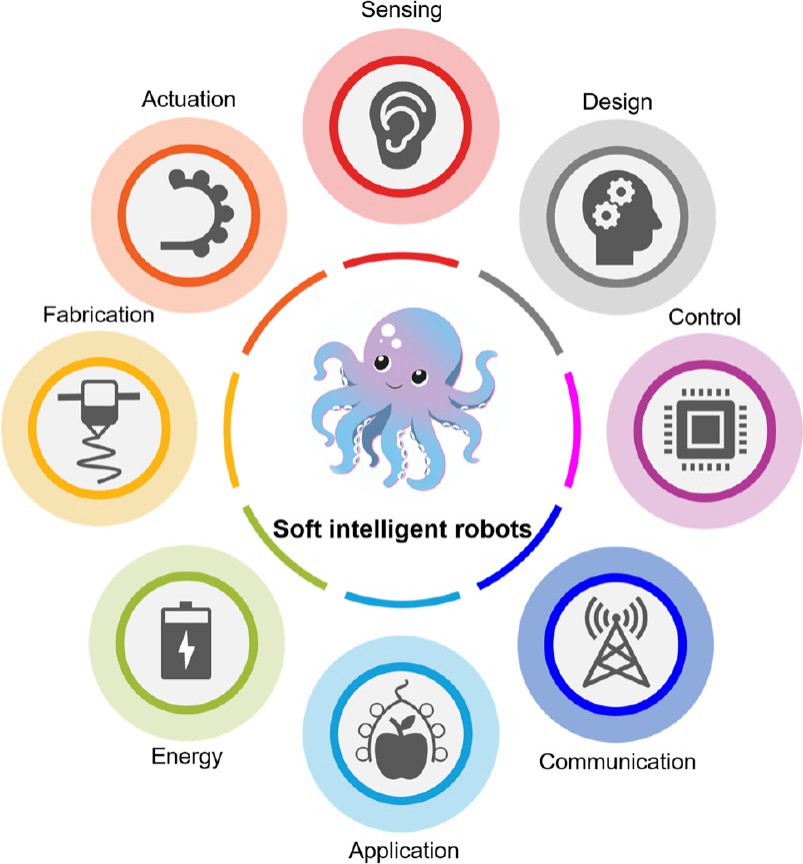
Components and considerations for the building of a smart soft robot. This figure highlights the key components essential for constructing a smart soft robot, including actuation, sensing, and energy/fuel for operation, as well as electronics for control and communication. Additionally, important considerations such as the fabrication method, design, control algorithms, and application aspects are depicted.

Before we go into sensing in soft robots, it is worthwhile to highlight the key components of a smart soft robot. A typical soft robot has several key components: (a) a soft body with soft functional organs, i.e., organs for locomotion and gripping; (b) embedded actuation mechanisms that might be soft or hard; (c) embedded sensors that provide the robot a sensory worldview of the surroundings; and (d) energy for functionality which might be stored on board or tethered to a stationary power source. In addition, depending on the complexity of a robot, there could be a rigid housing within a robot that safely houses all of the electronic controls and power source and acts as a power house and brain of the robot. Thus, it is essential to note that a smart soft robot comprises both soft and rigid parts which are intricately embedded to build a fully functional soft robot. Figure 1 illustrates various aspects to be considered for the design and manufacturing of a smart soft robot. Building a smart soft robot involves several important considerations: (a) choice of the right materials, (b) design that incorporates an actuation mechanism, electronics, sensors, communication, and energy source, (c) manufacturing methods, and (d) the algorithm for processing the sensor data and for robot control. The following section briefly discusses key actuation mechanisms used in soft robotics.

## Actuation Technologies for Soft Robotics

Like the muscles of human beings, soft actuators take the responsibility for robots to move, act, and perform given tasks. Actuation technology is regarded as one of the core challenges for soft robotic research.^19−21^ Different actuation mechanisms are critical for soft robotic system design in terms of fabrication, sensing, control, and working environments.^22−25^ Therefore, we start this review by introducing the most promising actuation mechanisms for soft robots.

### Fluidic Actuation

**Figure 2:**
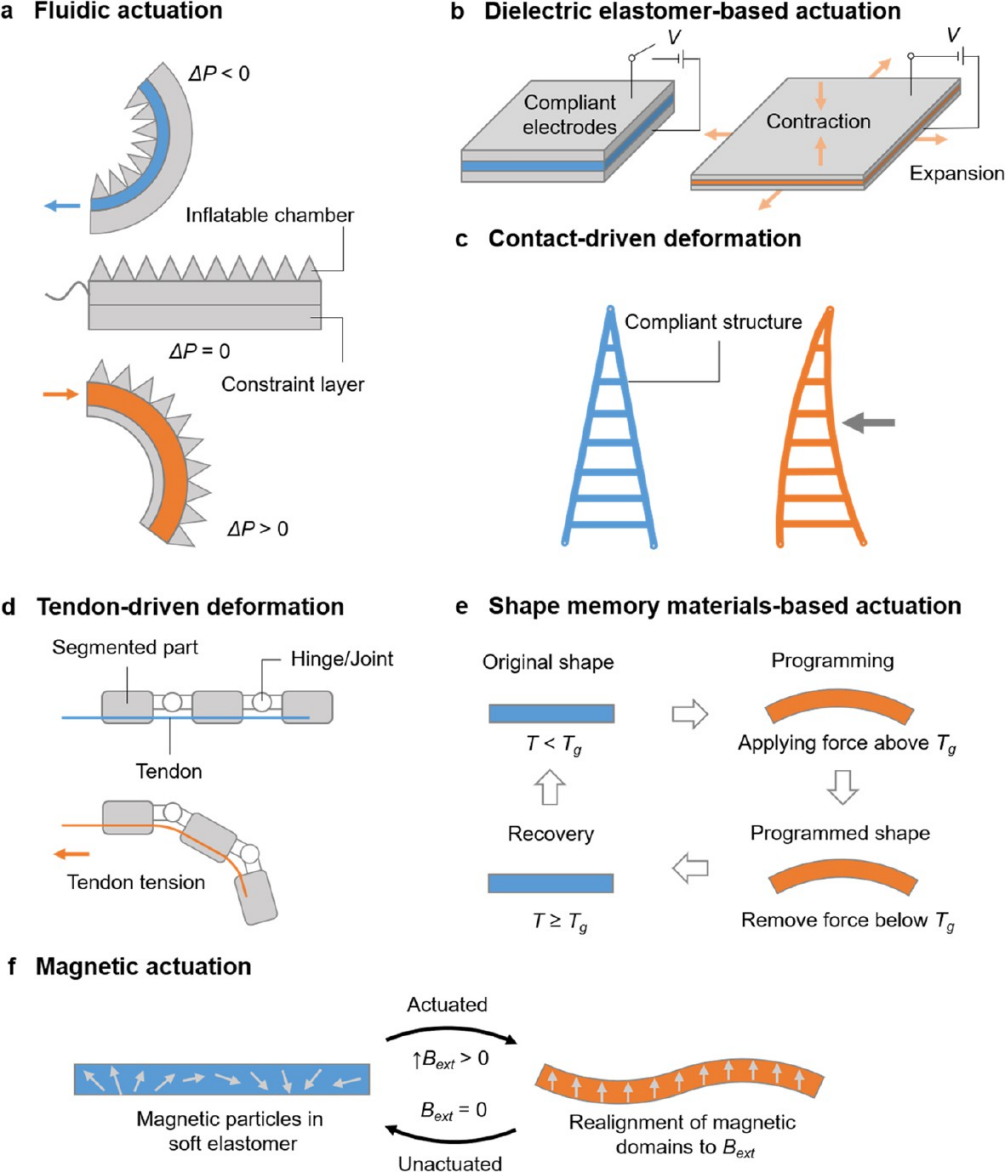
Actuation technologies for soft robotics. (a) Fluidic actuation. Actuation in soft robots is achieved by regulating the fluidic pressure *P* (positive or negative) within their internal inflatable cavities. The flow direction of the fluid, typically air or liquid, is indicated by arrows. (b) Dielectric elastomer-based actuation. Deformation of the sandwiched dielectric materials occurs as a result of attractive electrostatic forces between two compliant opposing electrodes. (c) Contact-driven deformation. External mechanical stimuli can cause the passive deformation of a compliant structure. (d) Tendon-driven deformation. When the tendon is pulled, the tendon tension leads to deformation of the gripper. The elastic hinges/joints can store bending energy, which enables the actuated fingers to return to their initial position. (e) Shape-memory materials based actuation. Actuation in shape-memory materials occurs when the temperature rises above a certain threshold for the shape-memory effect. (f) Magnetic actuation. Magnetic particles reorient along the direction of external magnetic field *B*_ext_ that causes the deformation of the soft elastomer. Gray arrows in the soft elastomer denote the orientation of the local magnetic domains.

Benefiting from the facile fabrication process, fast response time, tunable and wide range of gripping force, and low cost, fluidic actuation is one of the most widespread actuation mechanisms for soft robotics.^26−29^ As shown in Figure 2a, fluidic actuation achieves controllable movements through inflation or deflation inside a deformable chamber. Due to the asymmetrical design either in materials composition or structural geometry, soft grippers actuated by fluidics can bend toward two opposite directions under positive and negative pressures. Specifically, elastomeric materials are mostly employed to enable the deformation embedded with one or more inextensible layers to improve the stability and safety of the gripper. The actuation amplitude and rate of the soft robot are well-regulated by controlling the fluidic pressure and frequency of actuation media (air or liquid). This versatility of a soft robot could be used for a myriad of robotic grasping, locomotion, and wearable devices. However, there are also some limitations for fluidic-driven soft robots, such as bulky systems caused by the external pump to drive the actuation media and difficulty in accurate system control due to the nonlinear property of elastomeric materials.^30,31^

### Dielectric Elastomer-Based Actuation

Dielectric elastomer-based actuation is another widely used approach for soft robot actuation.^32−35^ As shown in Figure 2b, a thin elastomeric membrane is sandwiched between two compliant electrodes on which attractive electrostatic forces are applied for this actuation mechanism. When external voltages are applied on the two electrodes, they start to attract each other and the sandwiched elastomeric membrane is squeezed by Maxwell stress.^36^ The reported actuation voltages are dependent on the geometry, materials, and design of the soft robotic system. Generally speaking, dielectric elastomer-based actuation offers a wide range of advantages in terms of easy control, high power density, wide-range tunable stiffness and fast response speed, as the connection between the applied voltage and the elastomeric deformation is instantaneous and direct.^37^ Nevertheless, this actuation method not only suffers from dielectric breakdown caused by material defects but also often needs rigid frames to improve the output force, which may hinder the overall flexibility of the system.

### Contact-Driven Deformation

Inspired from the deformation of compliant structures, contact-driven deformation is a passive actuation method.^38−40^ Due to the special structural design, this kind of gripper can deform and conform to the surface of the grasped objects (Figure 2c). This passive adaptation is actuated by a servomotor that provides rotational or translational movements of the components. Thus, by controlling the servomotor, it is easy to control the contact-driven gripper to close, open, grasp, and hold an object.

### Tendon-Driven Actuation

Similar to the actuation principle by tendons in human fingers and other biological species, tendon-driven actuation provides another passive actuation solution for soft grippers.^41−44^ In this design, a cable or thread embedded in the soft gripper is connected with a servomotor (Figure 2d). The bending of the gripper can be accurately adjusted by controlling the servomotor, and corresponding bending energy is stored in the elastic hinges to allow the gripper to go back to the initial state.^45^ Like contact-driven and tendon-driven actuation methods, one possible challenge for soft robots actuated by motors lies in the miniaturization of the robotic system, as the design, mechanics, and control of the rigid and bulky motors are well-established.

### Shape Memory Materials Based Actuation

As one of the most popular smart materials, shape-memory materials provide another choice for the actuation of soft robots.^46−49^ Such materials can change from one shape to another shape under external stimuli (mostly by heat) due to the phase transformation of the materials (Figure 2e). The two most representative shape-memory materials are shape-memory polymers (SMPs) and shape-memory alloys (SMAs), and both exhibit stiffness variation under stimuli, which can be further employed for various kinds of robotics. The shape-memory effect in metals arises from a reversible phase transition between the martensite and austenite crystal structures,^50^ which can be induced by the application of heat and force. Conversely, in polymers, the shape-memory effect is achieved through a dual component system within the polymer. One component remains elastically deformable, while the other component can reversibly alter its stiffness when subjected to force and heat.^51^ The process is illustrated in Figure 2e. Initially, the shape-memory polymer (SMP) is subjected to a load at a temperature above its glass transition temperature (*T*_g_). Under these conditions, the polymer enters a soft rubbery state due to the increased mobility of the molecular chains. However, the applied force causes directed alignment of the molecular chains, leaving the material in a high-energy state. Subsequently, the SMP is cooled while maintaining the load, effectively “locking” the polymer in its programmed shape. Below the *T*_g_, the glassy phase restricts the movement of the molecular chains, effectively trapping them in the high-energy state.^52^ Upon reheating the SMP above its *T*_g_, the polymer softens once again and elastic recovery occurs as the molecular chains regain mobility, leading to the restoration of the original shape. Leveraging this shape-changing principle, shape memory can be programmed to provide a required actuation of specific shapes that could be achieved by 3D printing.^53−55^ Easy miniaturization and control are two merits for shape memory materials based actuation. Nevertheless, the actuation speed and hysteresis are the main challenges confronted by this actuation approach.

### Magnetic Actuation

A magnetic field can also be used to actuate a soft robot,^56−58^ and it has recently been intensively investigated (Figure 2f). The orientation of the magnetic domain is controlled or preprogrammed locally in the soft robot either by printing or microfabrication.^58^ Once the magnetized soft robot is placed in a magnetic field, it can move and be actuated by magnetic torques or forces and its movement mode can also be regulated by controlling the external field. With the rapid advancements in magnetic material design as well as in controlling the external magnetic field, a variety of complex movements are being achieved. The most fascinating advantages of magnetic actuation is that it allows untethered and relatively long distance control of soft robots, which is promising for robots working in confined environments such as inside the human body.^59,60^ However, for real healthcare applications for human beings, higher requirements are needed in electromagnetic setups in terms of control, power, and anti-interference. Nevertheless, advanced and sophisticated movement and functions of soft robots can be achieved by combining the merits of the above-mentioned actuation mechanism. Except for the above-mentioned actuation mechanisms, there are other actuation technologies for soft robotics, such as light, acoustic, temperature, chemical and biohybrid stimuli, etc.^61−67^

**Figure 3:**
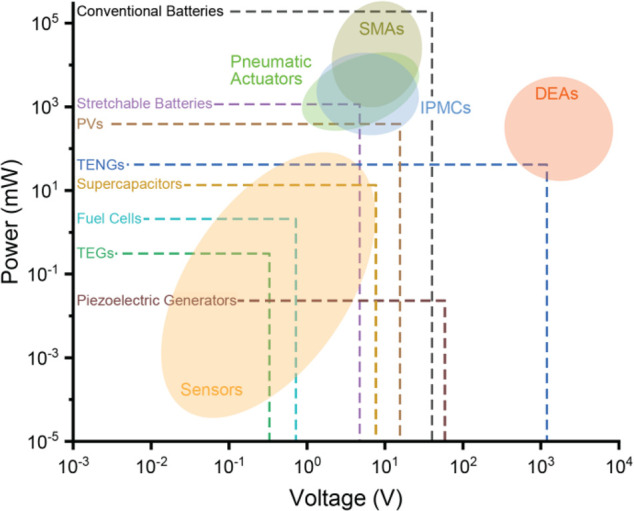
Comparison of power consumption of different actuation mechanisms typically used in soft robotics. Reproduced with permission from ref (68). Copyright 2022 The Authors under Creative Commons Attribution 4.0 International License, published by IOP Publishing.

In addition to exploring the various applications and advancements in soft robotics technology, an essential aspect of consideration is power requirements. Mazzolai et al. conducted a comparative analysis^68^ of power requirements (Figure 3) for different soft robotics and sensor systems. According to their findings, pneumatic actuators and ionic polymer–metal composites exhibit the lowest driving voltage, typically around ∼10^1^ V, with power consumption ranging from ∼10^2^ to 10^4^ mW. Shape-memory alloys also operate at a similar driving voltage (∼10^1^ V) but require higher power consumption, ranging from 10^3^ to 10^5^ mW. On the other hand, dielectric elastomer actuators demand the highest driving voltage, around ∼10^3^–10^4^ V, but consume relatively lower power, ranging from 10^1^ to 10^4^ mW compared to pneumatic actuators. It is worth noting that conventional batteries generally provide sufficient voltage and power to drive most soft robotic devices when operating independently. However, for dielectric elastomer actuators (DEAs), a step-up voltage regulator is required to achieve the necessary voltage levels. These power requirements have important implications for the design and implementation of soft robotics systems, especially on off-grip deployment.

In their study, Shintake et al. presented a comprehensive comparison^20^ of different types of soft actuators, highlighting their performance characteristics. The findings indicate that dielectric elastomer actuators (DEAs) exhibit the fastest response time, typically ranging from 0.1 to 1 s. When comparing ionic polymer–metal composites (IPMCs) to DEAs, DEAs outperform IPMCs in parameters such as the object mass/gripper mass, gripper size, and object size. The advantage of IPMCs over DEAs is primarily in terms of the driving voltage, as previously mentioned. On the other hand, fluid-driven elastomer actuators (FEAs) demonstrate the highest object mass/gripper mass ratio, allowing for handling heavier objects. The response time for FEAs varies from 0.1 to 6 s. Passive structures with external motors do not have a response time and provide an instant response. However, their overall performance is influenced by the design and material selection. Currently, shape-memory alloys (SMAs) may have limitations in all four comparison parameters. However, one noteworthy advantage of SMAs is their ability to retain deformation without requiring continuous energy input, unlike other methods that rely on mechanical locking mechanisms. These comparative assessments highlight the strengths and weaknesses of different soft actuator technologies, emphasizing the importance of considering specific performance parameters and requirements when selecting the most suitable actuator for a given application.

### Materials for Soft Robots

**Figure 4:**
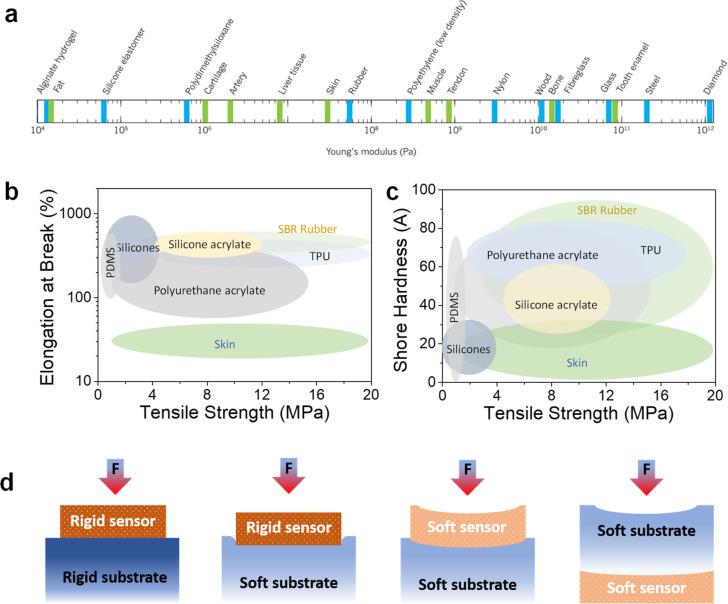
Material properties of the typical elastomers used for soft robotics. (a) Young’s moduli of various materials. Reproduced with permission from ref (77). Copyright 2015 Springer Nature. (b) Elongation at break and tensile strength of typical elastomers. (c) Comparison of shore hardness vs tensile strength of various classes of elastomers. Data collected from refs (78−96). (d) Different scenarios of the combination of sensor and substrate rigidities for integration of sensors in soft robots.

A fundamental criterion for a soft robot is to have a soft contact with the desired object; hence, the materials used for soft robotics are typically polymers that are stretchable, compressible, and flexible. Therefore, in the fabrication of a majority of the reported soft grippers, silicones, polyurethanes, gels, dielectric elastomer,^69^ Ecoflex,^56,57^ urethane rubber,^70^ and epoxy-urethane composite^71^ are used. The mechanical properties of these commonly reported materials are listed in Table 1 for reference. In addition, some of the soft robots have also been designed using shape-memory alloys, electroactive polymers, and polymers responsive to stimulus such as light, pH, and magnetism. Further, self-healing materials such as furan-maleimide polymer networks,^72^ gels like agarose/polyacrylamide gels,^73^ and liquid crystal polymer^74,75^ based soft robots also have attracted attention in recent years. The choice of these materials is primarily determined by Young’s modulus, elongation at break, and shore hardness, which are estimates of the stiffness, stretchability, and softness of these materials. The mechanical properties of these polymers in comparison with various materials are shown in Figure 4a–c. An ideal material for soft robots possesses high elongation at break, high tensile strength, and low shore hardness.

**Table 1:** List of Material Properties of Different Elastomers Used in Soft Robots

Material	Tensile Strength (MPa)	Elongation at Break (%)	Shore Hardness (A)	Method of Fabrication	Ref
Thermoplastic polyurethane	4–30	300–800	50–85	Injection molding, 3D printing	(78−80)
SBR rubber	3.4–20	450–600	30–95	Injection molding	(81, 82), https://rahco-rubber.com/materials/sbr-styrene-butadiene-rubber/
PDMS	0.1–5	100–500	10–70	Molding	(83, 84)
Ecoflex	1.3–4	300–900	0– 30	Molding	(85, 86), https://www.smooth-on.com/products/
UV curable polyurethane	0.8–10	100–500	20–40	3D printing	(87−90)
UV curable silicones	4–9	300–600	20– 60	3D printing	(91−93), https://www.protolabs.com/services/3d-printing/plastic/silicone/
Skin	0.1–20	10–80	0–30		(94−96)

Herein, the discussion related to materials used for soft grippers is very relevant with regard to tactile, force, and pressure sensing. The sensor’s location plays an essential role in determining the accuracy of the measured force. For instance, in the case of rigid robots, when the pressure sensor is located on the surface and comes into contact with the object of interest, the entire force of application is experienced at the sensor. In contrast, for a soft interface, the real force at the sensor depends on the relative location of the sensor with respect to the casing and the relative softness of the sensor with respect to the soft robot itself. Figure 4d illustrates the above point accurately. For instance, when a rigid sensor is mounted on the surface of a soft gripper, the force experienced by the sensor is less than the force on the object since a part of the applied force compresses the soft substrate.^76^ Further, when the soft sensor is placed on the soft substrate or the soft sensor is embedded inside the soft substrate, the sensor experiences lesser force than the force at the object. The sensor’s sensitivity is high if it is relatively softer than the substrate of the soft robot, wherein the sensor undergoes larger compression than the substrate. Therefore, the rigidity of the sensor, the substrate, and the object of interest all determine the accuracy of the force measurement. Thus, the material properties of the sensor and the substrate and the integration technique should be optimized for accurate force sensing. In the next section, we elaborate on various types of sensors used in soft robots.

## Sensing Technologies for Soft Robotics

Due to their inherent softness and mechanical compliance, soft robots are safer, more practical, and seamless for human–machine interactions and other applications compared with the conventional rigid ones. Except for actuation, sensing for soft robots is another grand challenge that must be addressed to achieve intelligent soft robots and complete specific tasks. For example, sensing for soft robots requires all of the electronic components, such as electrodes, sensors, and encapsulation layer on the soft robotic body, to be flexible or even stretchable, which is in great contrast with the conventional hard and rigid silicon-based electronics. Thanks to the rapid advancements of artificial skin and flexible electronics in recent years, sensing for soft robots has become possible. Compared with rigid silicon-based electronics, flexible electronics aims to develop an alternate electronic paradigm^97^ by employing intrinsically conductive materials or designing special structures to fabricate flexible and stretchable functional devices.^2,98,99^ There are a lot of research works about artificial skins based on resistive,^100^ capacitive,^101^ triboelectric,^102^ magnetic,^103^ optical sensors^104^ that exhibit excellent sensing abilities. In the following part, several of the most prevalent sensing mechanisms for soft robots will be introduced and discussed.

### Resistive Sensors and Piezoresistive Sensors

Both resistive and piezoresistive sensors use resistance as the indicator of the variation of pressure or strain resulting from external stimuli. The resistance of a material follows Ohm’s law, which can be written as follows:
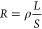
where *R* denotes the resistance of a material, ρ the resistivity, *L* the length, and *S* the cross-section area. According to this formula, the electrical resistance of materials is related with the resistivity, length, and cross-sectional area of materials. These dictating parameters are dependent on the deformation of the materials. Therefore, resistive and piezoresistive sensors can indicate the bending state or external pressure applied to the soft robotic body by the variation of resistance. In other words, these sensors not only allow soft robots to gain more tactile information when they have contact with the external environments but also enable their proprioception by measuring the bending state of the robot.

**Figure 5:**
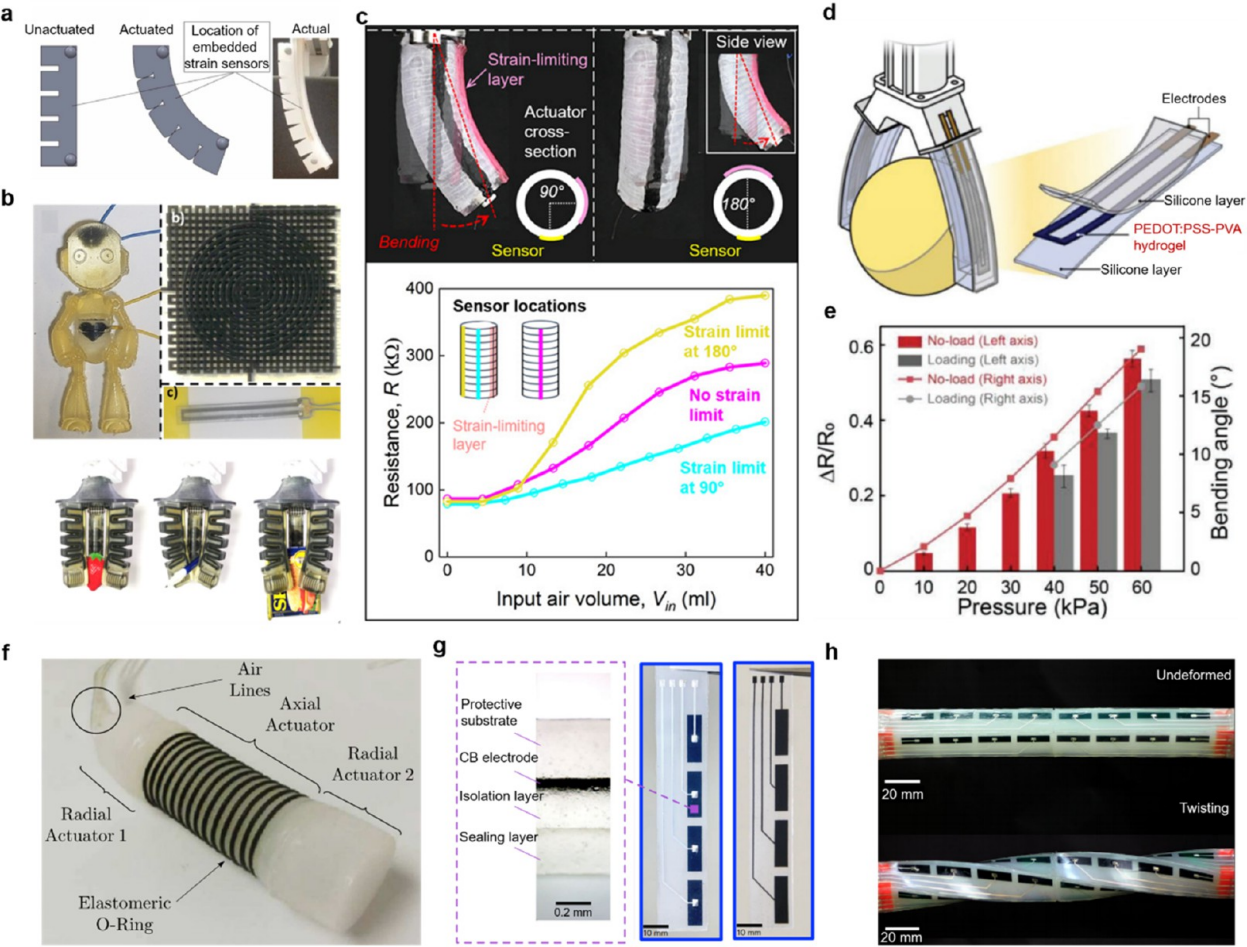
Resistive, piezoresistive, and capacitive sensors for soft robots. (a) Schematic illustration and photograph of soft actuator with infrared-reflective balls for motion-tracking the motion and embedded sensors for estimating the contact forces. Reproduced with permission from ref (16). Copyright 2019 American Association for the Advancement of Science. (b) Top: Humanoid robot and a corresponding multilayer strain and pressure sensor. Bottom: Soft gripper with embedded resistive sensors grasping three different kinds of objects. Reproduced with permission from ref (105). Copyright 2019 The Authors under Creative Commons Attribution License (CC BY), published by Frontiers. (c) Top: Printed composites on a fiber-reinforced actuator as strain sensor at different locations. Bottom: Electrical resistance variation of three strain sensors as a function of the input air volume. Reproduced with permission from ref (106). Copyright 2020 American Association for the Advancement of Science. (d) Schematic illustration of a soft robotic machine integrated with a PEDOT:PSS-PVA hydrogel strain sensor. (e) Relative changes in resistance and bending angles of the sensory gripper under loading and unloading conditions, when pneumatic pressure increases from 0 to 60 kPa. Panels d and e reproduced with permission from ref (107). Copyright 2022 Wiley-VCH GmbH. (f) Earthworm-like soft robot, which consists of several actuators and soft skin sensors distributed on two ends. Reproduced with permission from ref (108). Copyright 2019 IOP Publishing. (g) Components (left) and photograph (right) of the intrinsically stretchable capacitive e-skin for soft robot. (h) Picture of the soft arm in undeformed (top) and twisting (bottom) states, integrated with stretchable capacitive e-skin for high-resolution morphological reconstruction. Panels g and h reproduced with permission from ref (109). Copyright 2023 Springer Nature.

By integrating strain and pressure sensors on a soft actuator, Farrow and Correl^5^ demonstrated that the combination of these sensors can enable proprioception of both contact forces and the bending state of the gripper. This soft actuator can also be used for detecting unexpected contact with the surroundings as well as grasp failures of objects. To reduce the fabrication complexity, Bilodeau et al. used liquid metal as strain sensors^110^ for a soft gripper, and the liquid metal was embedded in the fluidic channel of the gripper. This sensor demonstrated repeatable performance when the gripper was actuated and could be used to provide real-time feedback for the robotic system. Although the resistive sensor can provide a solution for soft robot proprioception, soft robotic systems exhibit nonlinear behavior, which makes them hard to model and predict. To address this, Thuruthel et al.^16^ developed a system for soft robot perception by combination of embedded sensors, a vision-based motion capture system, and a machine learning approach to model an unknown soft actuated system successfully. As shown in Figure 5a, the soft resistive strain sensors were embedded in the soft actuators for estimating the coordinates of the tip as well as the forces generated by the actuator. The sensory information from the sensors and the marker information from the motion capture system were required in a variety of configurations. This approach has been proven valuable for real-time modeling of the kinematics of soft continuum actuators, demonstrating robustness against sensor nonlinearities and drift. This approach grasps inspiration from the human perceptive system, in terms of hands and eyes, which is promising for applications such as human–robot interaction and soft orthotics by providing a more accurate force and deformation model. To improve the seamless integration of sensors with soft robots, Shih et al. used multimaterial 3D printing technology to fabricate soft robots with sensing abilities without additional processes.^105^ As shown in Figure 5b, the printed resistive and soft sensors can be integrated into both a humanoid robot and a soft gripper, which can be used to differentiate different objects. Typically, soft conductive materials are composed of conductive fillers and polymer matrices. This conductive composite material is regarded as one of the candidates for soft robotic sensing materials. However, during preparation of the composite materials, the solvents may cause swelling and decomposition of the polymer substrate, which greatly hinders the application of the composite materials. To address this issue, Kim et al.^106^ reported the ethanol-based Pickering emulsion approach to manufacturing conductive composites. This safe and sustainable fabrication approach for soft conductive composites is compatible with a variety of substrates and also printing technology. As shown in Figure 5c, the composites are directly printed on a soft actuator as strain sensors. Benefiting from this, the motion of the actuator can be tracked.

Koivikko et al.^111^ employed screen printing as another scalable technology to manufacture sensors for soft robots. Resistive curvature sensor was printed with silver inks and then integrated into soft pneumatic grippers. The sensor exhibited a linear relationship between the bending curvature and resistance; however, the hysteresis is 17%. Unfortunately, many of these strain sensors have a large hysteresis and low stretchability, which may limit their applications. Shen et al.^107^ reported an ultralow hysteresis (<1.5%) and stretchable (300%) hydrogel strain sensor composed of poly(3,4-ethylenedioxythiophene):polystyrene sulfonate (PEDOT:PSS) nanofibers and poly(vinyl alcohol) (PVA) (Figure 5d). The resistance of the hydrogel sensor increased linearly with the applied air pressure into the soft gripper, and its loading and unloading performance of the gripper can also be detected, as shown in Figure 5e. Calderon et al.^108^ demonstrated an interesting earthworm-inspired soft robot with sensing abilities (Figure 5f). In this scenario, by utilization of a combination of two radial actuators and a centrally positioned axial oscillatory mechanism driven by pneumatic force, the movement modes of an earthworm in terms of horizontal and vertical locomotion can be well-imitated. The sensing skins for this earthworm-inspired soft robot are made of deformable microchannels filled with conductive liquid metal eutectic alloys. The perceptive soft robot is able to measure strain and detect pressure variations in the surroundings. This provides convincing evidence that the approaches employed in this work for actuation, sensing, and control can facilitate the construction of extensive, intricate structures composed of fine modules for the development of autonomous intelligent soft robots.

### Capacitive Sensors

Capacitive sensors offer another option to measure the pose of soft robots as well as their interaction with the environment by measuring the change in capacitance. To develop flexible capacitive sensors, several stretchable and conductive materials are used to fabricate electrodes, such as composites,^112^ conductive polymer,^113^ and thin films.^114^ Another indicator of the performance of capacitive sensors is sensitivity, which is dominated by deformation of the dielectric layers. In order to increase the sensitivity of the sensor, a number of strategies have been utilized, ranging from use of porous structure,^115^ engineered surface,^116^ fabrics,^117^ and nanowires network.^118^ Moreover, the trade-off between the sensitivity and pressure range is another roadblock for soft capacitive sensors. To overcome this challenge, Ha et al.^119^ fabricated a hybrid piezoresistive and piezocapacitive sensor with high sensitivity over a wide range of pressure, which is promising for precise controlling of robots. While capacitive sensors have a large dynamic range, fast response, an excellent linear range, and sensitivity, they also have some drawbacks, such as susceptibility to contaminants, proximity effects, and sensitivity to mechanical perturbation. For example, due to the unavoidable coupling of mechanical deformation within the structure, there are variations of quantitative pressure measurement when the sensor is under stretching. This limitation for the sensing performance of the flexible sensor can hinder further application of the sensors for precise and quantitative detection of external pressure under deformation. Based on this, Su et al.^120^ reported a stretchable sensor that is insensitive to stretch, and this excellent characteristic was attributed to the synergistic combination of a pyramid microstructure with hierarchical stiffness distribution and electrical double-layer-based interfacial capacitive sensing mechanism. Hu et al.^109^ reported a technique for high-resolution morphological reconstruction for soft robots based on stretchable capacitive sensors. As shown in Figure 5g, this intrinsically stretchable capacitive sensor was composed of several layers: protective substrate, electrode layer, isolation layer, and sealing layer. After integration with a number of sensors on the soft gripper, the signals from the e-skin can be transformed to high-density point clouds that can accurately reflect the geometry of the gripper via machine learning technique (Figure 5h), which is of importance for solving fundamental problems in soft robotics, such as precise closed-loop control and digital twin modeling.

### Optical Sensors

Another interesting class of sensors that has seen increasing usage in soft robotics is optoelectronic-based sensors. Optoelectronic sensors show high sensitivity and fast response rate, accommodate noncontact sensing, have low power consumption, exhibit lower hysteresis, are immune to electromagnetic interference, and are resistant to chemical corrosion. In addition, by utilizing flexible and stretchable optical fibers inside soft robots, sensors can be easily integrated within soft robots.

**Figure 6:**
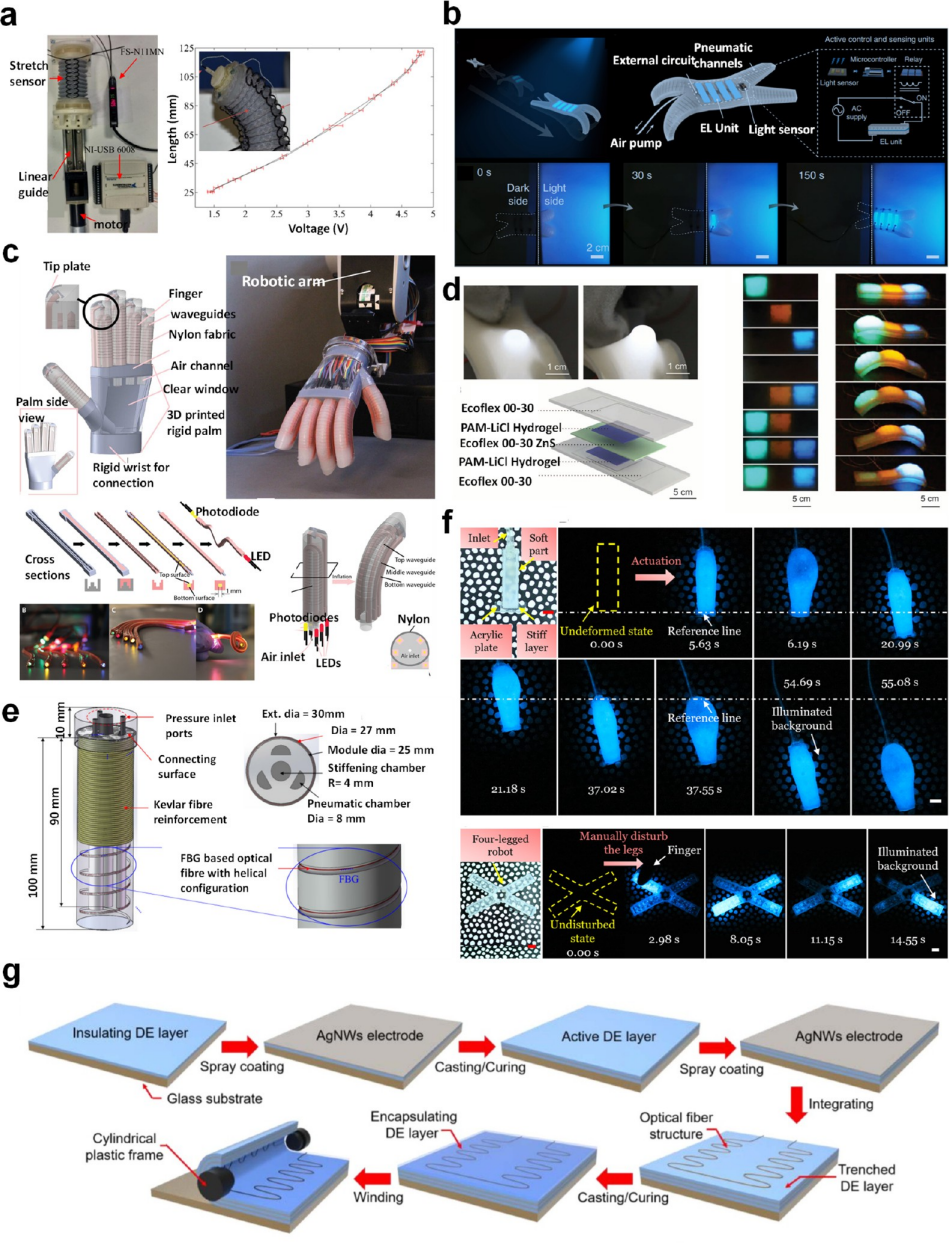
Optical sensors for soft robotics. (a) Left: Soft arm with sewn optical fibers as a macrobend strain sensor for pose measurement. Right: Voltage vs length relationship of the stretch sensor. Reproduced with permission from ref (121). Copyright 2015 IOP Publishing. (b) Soft camouflage quadrupedal robot. Top: Design of the soft robot with integration of the sensors and EL skin. Bottom: Camouflage action of the robot during its motion by selective lighting of the EL skin on the soft robot. Reproduced from ref (122). Copyright 2022 The Authors under Creative Commons Attribution 4.0 International License, published by Springer Nature. (c) Soft robotic prosthetic hand embedded with stretchable waveguides for proprioception. Top left: Design of the gripper. Top right: Gripper with fully integrated sensors mounted on a robot. Bottom left: Method of fabrication of waveguides. Bottom right: Illustration showing the location of multiple waveguides for smart tactile sensing. Reproduced with permission from ref (123). Copyright 2016 American Association for the Advancement of Science. (d) Stretchable EL skin for tactile sensing. Left: Enhanced illumination of the EL skin under stretching. Bottom: Architecture of the EL skin. Right: Selective illumination of the deformed parts of a soft robot by the EL skin. Reproduced with permission from ref (113). Copyright 2016 American Association for the Advancement of Science. (e) Soft surgical robot with helically embedded fiber Bragg grating based optical fiber. Reproduced with permission from ref (124). Copyright 2021 The Authors under Creative Commons Attribution 4.0 International License, published by Optica Applicata. (f) Electronics free biohybrid mechanoluminescent soft robot. The illumination is induced by both mechanical perturbation around the robot and actuation itself. Top: Undeformed and actuated states observed at different times. Bottom: Illumination state of the quadruple robot after mechanical disturbance at different times. Reproduced with permission from ref (125). Copyright 2022 The Authors under Creative Commons Attribution 4.0 International License, published by Springer Nature. (g) Illustration of method of fabrication of thermoformed optical fiber embedded within a dielectric elastomer capable of strain measurement. Reproduced with permission from ref (126). Copyright 2021 IOP Publishing.

For instance, Song’s group demonstrated an omniadaptive soft gripper^127^ with embedded optical fibers for tactile sensing. The optical fibers were inserted inside the structural cavity of the finger without interfering with its adaptive performance. The smart grippers could sort the object’s dimensions within ±6 mm error and measure the structural strains within ±0.1 mm. The researchers used the commercial optical fiber comprising a poly(methyl methacrylate) (PMMA) core (2 mm diameter) and transparent polytetrafluoroethylene (PTFE) clad with a low attenuation loss of 0.2–0.5 dB/m. The sensing was accomplished by measuring the change in voltage signal of the photoresistor at the end of the optical fiber due to the deformation of the beams of the finger during the gripping action. Another interesting approach is to utilize fiber Bragg grazing (FBG) marked optical fibers inside a gripper for sensing deformation. The deformation causes a shift in the wavelength of the transmitted light, which can be correlated to the deformation for accurate estimation of bending, compression, or stretching movements. In another report, Althoefer’s group^121^ used macrobend optical sensors for pose measurement of the soft robotic arm. Here, the macrobend stretch sensor is an optical fiber that modulates the intensity of the transmitted light due to bend, stretch, and compression force. Three macrobend sensors (Figure 6a) were sewn along the periphery of the soft arm with an equal orientation of 120° from each other. The sensor could accurately distinguish between bend, stretch, and compression of the arm based on the change in intensity due to transmission loss at the macrobends. Electroluminescence is another class of optical-sensing mechanisms that can be incorporated into soft robots’ sensing. Liu’s group^122^ fabricated a soft quadrupedal robot with an electroluminescent (EL) layer that has the capability to camouflage the surface of the robot with three blue, green, and orange-colored backgrounds. As shown in Figure 6b, a light sensor is mounted on the quadrupedal robot, which senses the wavelength of the ambient light and triggers the lighting of a respective layer of EL material. The smart soft robot was fabricated by multimaterial 3D printing of in-house-developed ion-conducting, electroluminescent, and dielectric inks, which enabled the fabrication of such a complex architecture. Shepherd’s group has reported several studies incorporating optoelectronic sensors in soft robots. For instance, stretchable waveguides were fabricated using transparent polyurethane rubber (VytaFlex 20, Smooth-On Inc., η = 1.461, 2 dB/cm) as a core and silicone composite (ELASTOSIL M 4601 A/B, Wacker Chemie AG, η = 1.389, 1500 dB/cm) as a cladding material.^123^ The stretchable waveguides (Figure 6c) were embedded in a soft robotic gripper for measuring the force, roughness, shape of the surface, and softness of the objects. Their sensing mechanism relied on the loss of optical signal due to bending during actuation and on compression of the waveguide with the compression force. By using multiple waveguides within each gripper, it was possible to accurately analyze the optical signal arising from bending and compression force separately. The grippers with the embedded sensors were fabricated by the multistep casting of elastomers within 3D-printed molds. Shepherd’s group also fabricated an electroluminescent skin^113^ for tactile sensing. As shown in Figure 6d, EL skin was fabricated by sandwiching an electroluminescent ZnS–silicone composite between hydrogel electrodes and finally encapsulated them within a silicone elastomer. The relative illumination of the EL layer and the capacitance of the electrodes changed with different degrees of elongation. This changes in capacitance and illumination intensity were used to monitor the stretching, folding, and rolling of the robot. Interestingly, the stretching of the elastomer causes localized illumination of the EL layer, providing a visual cue for the location of the deformation of the robot, which is useful in the design optimization of soft actuators. Xu’s group fabricated a soft surgical robot^124^ with FBG-based optical fiber embedded in a spiral fashion, as shown in Figure 6e. The helical configuration prevented the dislocation of sensors during actuation and supported material stretchability in contrast to mounting in a linear fashion. The FBG optical fiber sensed the bending movement within a 2.5% error. The soft surgical tool was fabricated by casting Ecoflex-00 inside a 3D-printed mold with helical grooves. The FBG optical fiber was placed along the grooves, and a second layer of Ecoflex was cast on it to anchor the optical fiber strongly. The FBG grating length was 10 mm, with a bandwidth of 10 dB, with a center wavelength ∼ 1535 nm. In another report, Cai’s group developed power-free soft biohybrid mechanoluminescent^125^ soft robots. The mechanoluminescence was achieved by encapsulating dinoflagellates, bioluminescent unicellular marine algae, within the chambers of the soft actuators. The observed ML was nearly instantaneous, with the ability of light emission maintained over weeks without special maintenance. As shown in Figure 6f, the device was demonstrated for its usefulness in the visualization of external mechanical stimuli, deformation-induced illumination, and optical signaling in dark environments. The key characteristic of their research was composed of using bioluminescent materials, which reduces the complexity of the electrical circuitry of the EL devices.

In another report by Kyung’s group,^126^ the optical strain sensor was fabricated by thermoforming a polymer optic fiber in a bitortuous structure inside a silicone elastomer. They used commercial optical fiber (SH1001-ND EskaTM) with PMMA core (240 μm; refractive index, 1.49) and F-doped PMMA (5 μm; refractive index, 1.41) as a clad. As shown in Figure 6g, the sensor benefits from the curvilinear design of the optical fiber inside the elastomeric structure, enabling strain measurement in a wide range from 0 to 120%. The strain sensing is fast and reversible with a small hysteresis even under cyclic loading. The sensor was successfully used to monitor the dynamic deformation of the soft actuator. In another report by Shepherd’s group, they embedded optical fibers^128^ inside the fingers of a soft gripper with interconnected pressure chambers. The clad of the optical fibers was removed strategically at fixed places using a laser engraver to facilitate losses during bending. The sensor could accurately predict the bending motion of the fingers during actuation, which was used for closed-loop soft orthosis. In another exciting approach, Park’s group^129^ fabricated soft grippers with stretchable waveguides with a reflective metal coating. Silver-coated waveguides were prepared by layer transfer deposition (LTD) of silver on a 3D-printed mold, followed by casting the silicone elastomer on to the silver-coated mold. When the elastomer is peeled off after curing, the metal layer is transferred to the elastomer, creating a reflective coating. The light is transmitted almost fully under unstretched conditions due to total internal reflection. However, microcracks are formed on the waveguide under stretched condition, which reduces the transmitted light intensity, facilitating the sensing mechanism. The sensor exhibited high compliance and low hysteresis, which were used to accurately control the bending curvature of the sensor during the gripping of objects.

In the next section, we describe various other types of sensing mechanisms that have been reported for soft robots.

### Other Sensing Methods

**Figure 7:**
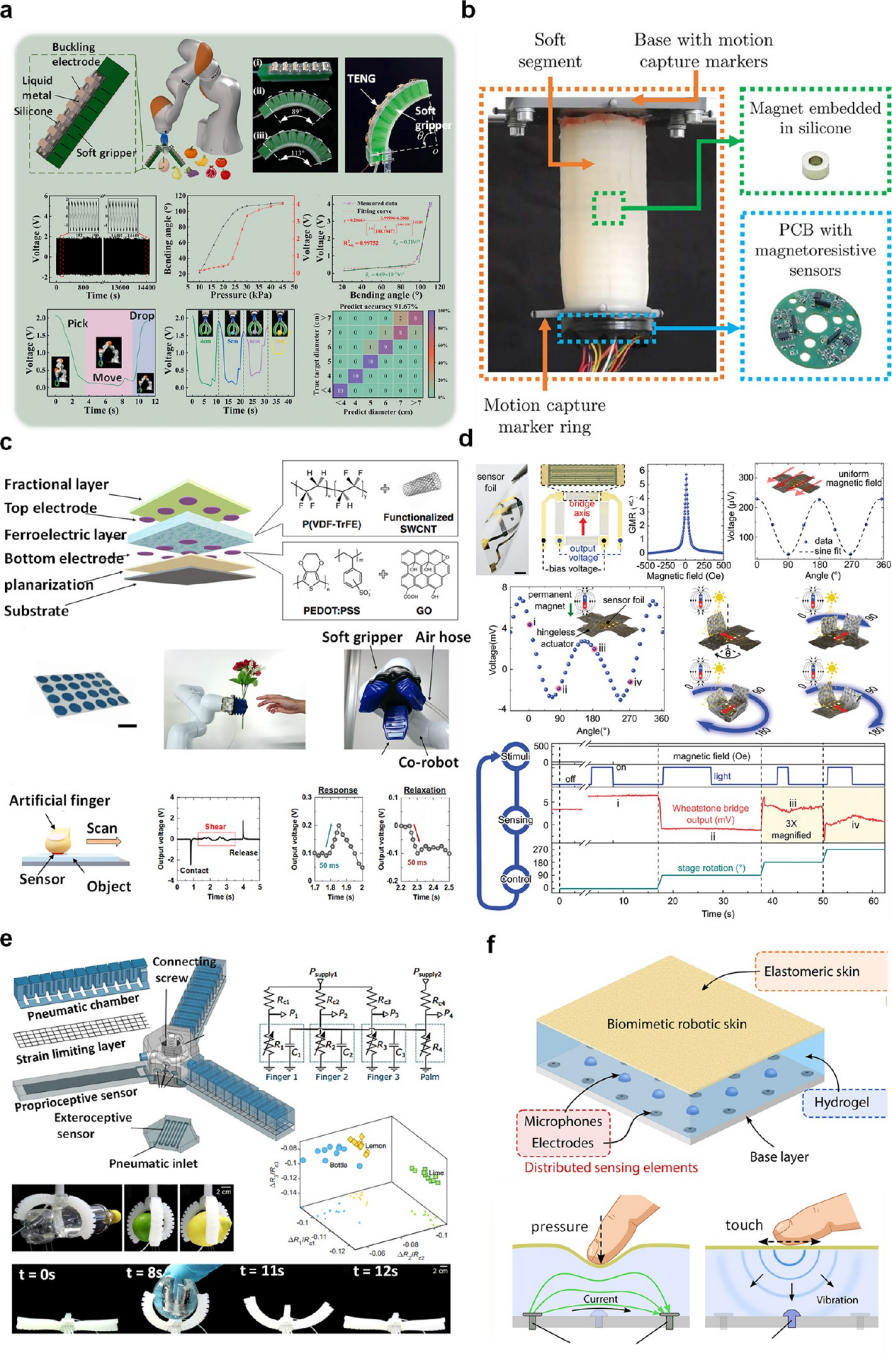
Various other types of sensors used for soft robotics. (a) Soft gripper with triboelectric nanogenerator (TENG) sensors for shape and size sensing. Top: Schematics and fabricated soft gripper with integrated TENG sensors. Middle: Variation of voltage signal at the sensor for different input pressure/bending angle and cyclic tests. Bottom: Variation of the sensor’s voltage signal during gripping of the items of different dimensions and confusion map of object diameter recognition using the sensor. Reproduced with permission from ref (130). Copyright 2022 Elsevier. (b) Soft robotic arm with embedded magnet and magnetoresistive sensor for controlling the arm position. Reproduced with permission from ref (133).Copyright 2022 The Authors under Creative Commons Attribution 3.0 Unported License, published by Royal Society of Chemistry. (c) Soft gripper with highly sensitive slip sensor using ferroelectric polymer and nanocarbon. Top: Exploded view of the architecture of the sensor. Middle: Integration of the sensor on the soft gripper. Bottom: Sensor data measured in voltage under contact, shear, and release conditions. Reproduced with permission from ref (134). Copyright 2021 The Authors under Creative Commons CC BY License, published by Wiley-VCH GmbH. (d) Origami grippers with the ability to sense bend and rotation using a magnetic polymer composite. Top left: Picture of sensor foil. Top center: Angle sensor based on four sensors connected in wheat stone bridge configuration and the corresponding sensor data. Middle: Output voltage of the wheat stone bridge as a function of angle of magnetic field and the assembly steps for folding the magnetic origami into a flower. Bottom: Feedback loop for the controlled folding of the origami. Stimuli come from a magnetic field and light, which are detected by the sensor. The sensor data are used to control the rotation of the stage. Reproduced with permission from ref (135). Copyright 2021 The Authors under Creative Commons CC BY License, published by Wiley-VCH GmbH. (e) Soft gripper with pneumatic resistor and pneumatic strain gauge for electronics free tactile sensing. Top left: Schematic of the soft gripper with location of proprioceptive and exteroceptive sensor. Top right: Electrical equivalent of the pneumatic circuit—capacitors actuators, resistors–pneumatic restrictors, variable resistor–pneumatic strain gauge. Middle: Variation of the pneumatic resistor gauge signal while grasping different objects. Bottom: Movement of the gripper during actuation and grasping. Reproduced with permission from ref (136). Copyright 2022 The Authors under Creative Commons Attribution 4.0 International License, published by Springer Nature. (f) Biomimetic robotic skin capable of sensing both touch and pressure by a combination of microphones and ionic hydrogel. Top: Architecture of the biomimetic skin. Bottom: Compliant and electrically conductive hydrogel used as a pressure sensor and the embedded acoustic sensor for detecting light touch. Reproduced with permission from ref (137). Copyright 2022 American Association for the Advancement of Science.

Triboelectric nanogenerators are an interesting class of sensors which convert mechanical energy into electrical signals and therefore have been extensively used for sensing in soft robotics. Yang’s group^130^ demonstrated fabrication of a multifunctional sensor based on a triboelectric nanogenerator for a variety of applications: (a) detection and control of grasping (Figure 7a), (b) 2D motion of the robot, and (c) adaptive obstacle avoidance during soft robot locomotion. The soft gripper with a TENG sensor can detect the bending angle of the soft gripper which was used to estimate the dimensions of the object with an accuracy of ∼92%. Further, with smart arrangement of buckling electrodes, they achieved a 2D spatial decoupling structure capable of detecting movement direction and real-time position with an accuracy of ∼83%. The fabrication of the soft robot with integrated sensors was achieved by the assembly of the soft robot and the TENG sensor. Both the soft robot and the TENG sensors were fabricated by casting of silicone elastomer inside the molds. Recently, soft robotic grippers have been increasingly used for harvesting agricultural products. Such applications would need smart decisions regarding the maturity of the fruits and vegetables for harvesting. Hu’s group^131^ developed a soft gripper with a fruit ripeness sensor for detecting ripeness of blackberry. The tendon-driven gripper is coupled with a near-infrared (NIR) fruit ripeness detector that relies on reflectance modality to estimate the ripeness of the fruit. The gripper also comes with an endoscopic camera for visual observation. The average measured reflectance of ripe fruit (16.78) and unripe fruit (21.70) falls into two distinct regions for accurate estimation of ripeness during the harvest. In another report, Iida’s group demonstrated^132^ soft grippers mounted with electrical impedance-tomography-based sensors to estimate various characteristics of the fruit, such as weight, ripeness, and acidity (pH). The sensor works on the principle of bioimpedance, i.e., when an alternating current applied across organic matter biological tissue impedes the flow of current. The impedance is a function of the anisotropic composition of the material, which provides an estimate of the sugar content and acidity of the fruit. The pH and sugar content of sample fruits were measured to train the algorithm and later used to estimate the sugar content and acidity of the fruits close to the industrial tolerance limit of ±2 g for weight, ±0.2% for sugar content, and ±0.2 for pH. Baaij et al. fabricated a soft robotic arm equipped with magnetoresistive sensors^133^ (Figure 7b) and ring magnets within the robot that can estimate the shape of the actuator. They utilized the combination of the kinematic model of the magnetic sensors and the neural network to train the algorithm in estimating the accurate position of each segment of the robot. In another interesting work, Sekine et al. fabricated a slip sensor^134^ for soft robots using ferroelectric polymer with nanocarbon materials. By using nanocarbons and controlling the annealing process, they were able to rearrange the crystallinity of the sensing layer, which enabled ferroelectricity beyond 11.0 μC cm^–2^ and to detect a high acceleration value of 4.0 dV ds^–1^ with an applied force speed of 200 mm s^–1^. The sensor was mounted on a soft gripper (Figure 7c) to pick fragile objects with precise control of force by implementing a feedback-loop control using the sensor data. In another report of a magnetic sensor, Ha et al. designed a magnetic origami actuator^135^ capable of monitoring its own orientation and displacement along with the magnetization state. The origami grippers (Figure 7d) were fabricated from magnetic NdFeB microparticle embedded shape-memory polymers. The magnetoresistive electronic skins were laminated on the surface of the actuator for sensor feedback. An external magnetic field was used to actuate the origami structures, which was sensed by the magnetoresistive sensor. The sensor data were used for precise control of the magnetic field to achieve the appropriate fold and rotation of the origami structures to achieve a soft gripper with feedback control.

Since the majority of the soft grippers are actuated by pneumatic pressure, it is convenient to achieve feedback control by control of the pneumatic pressure. Koivikko et al. fabricated a soft gripper with pneumatic strain gauges^136^ free of electronics. The pressure sensor consists of a pneumatic chamber embedded within a silicone elastomer that acts as a pneumatic strain gauge that can measure up to 300% strain and show good stability without hysteresis. The key characteristic of this smart gripper is that the four major components (Figure 7e) of the robot, viz., actuators, logic, sensing, and power, are all pneumatic, enabling an electronics-free soft robot capable of proprioception and exteroception. In another approach, Park et al. fabricated biomimetic robotic skin^137^ enabled by tomographic imaging and hydrogel elastomer hybrids. The sophisticated sensor architecture (Figure 7f) consists of a tough hydrogel encapsulated within silicone elastomer, mimicking skin and the internal tissue of the human hand. The ionic hydrogel undergoes a change in conductivity under applied force, which is used to estimate the exerted force on the object. Further, the microphones under the hydrogel are sensitive to vibration which can detect a gentle touch on the surface of the robotic skin. The signals from the microphone and ionic hydrogel are relayed to deep neural networks which analyze the signal to accurately estimate the location and magnitude of the touching force on the robotic skin, bringing about high-accuracy (98.7%) touch measurement. Another interesting approach to sensing is the integration of sensors on the fabrics. The sensing materials are coated on the fibers which can be knitted into fabrics and integrated with soft robots for mechanical, humidity, and temperature sensing.^138^

In the next section, sensors that can sense multiple parameters for multimodal sensing and control of soft robotic devices are discussed.

### Integrated Sensors on Soft Robot for Multimodal Sensing

Tactile perception includes more than one piece of information. For example, there are pressure, temperature, vibration, and many other receptors on human fingers to guarantee the dexterity of hands. For instance, there are four types of mechanoreceptors distributed throughout the human body that can measure external forces on different time and space scales: two slow-adapting receptors (SA-I and SA-II) and two fast-adapting receptors (FA-I and FA-II). While the former two respond to static pressures and skin stretch, the latter two types measure object slip, edges, fine features, and vibrations. Benefiting from the collaborative work of these mechanoreceptors, human beings can discriminate a huge number of objects dexterously and accurately.

Like human skin, multimodal tactile perception is also vital for robots by providing them with the ability to interact with their surroundings precisely, rapidly, and safely. Recently, several research works about multimodal sensors that integrate different sensing modules into a sensing platform have been reported.^144,145^ After integrating these multisensory electronic skins on robots, their performance improved significantly in terms of objects manipulation and recognition,^146−148^ which provides foundation for development of intelligent robots. In the following section, several examples of soft robots integrated with multimodal sensors are discussed in detail.

**Figure 8:**
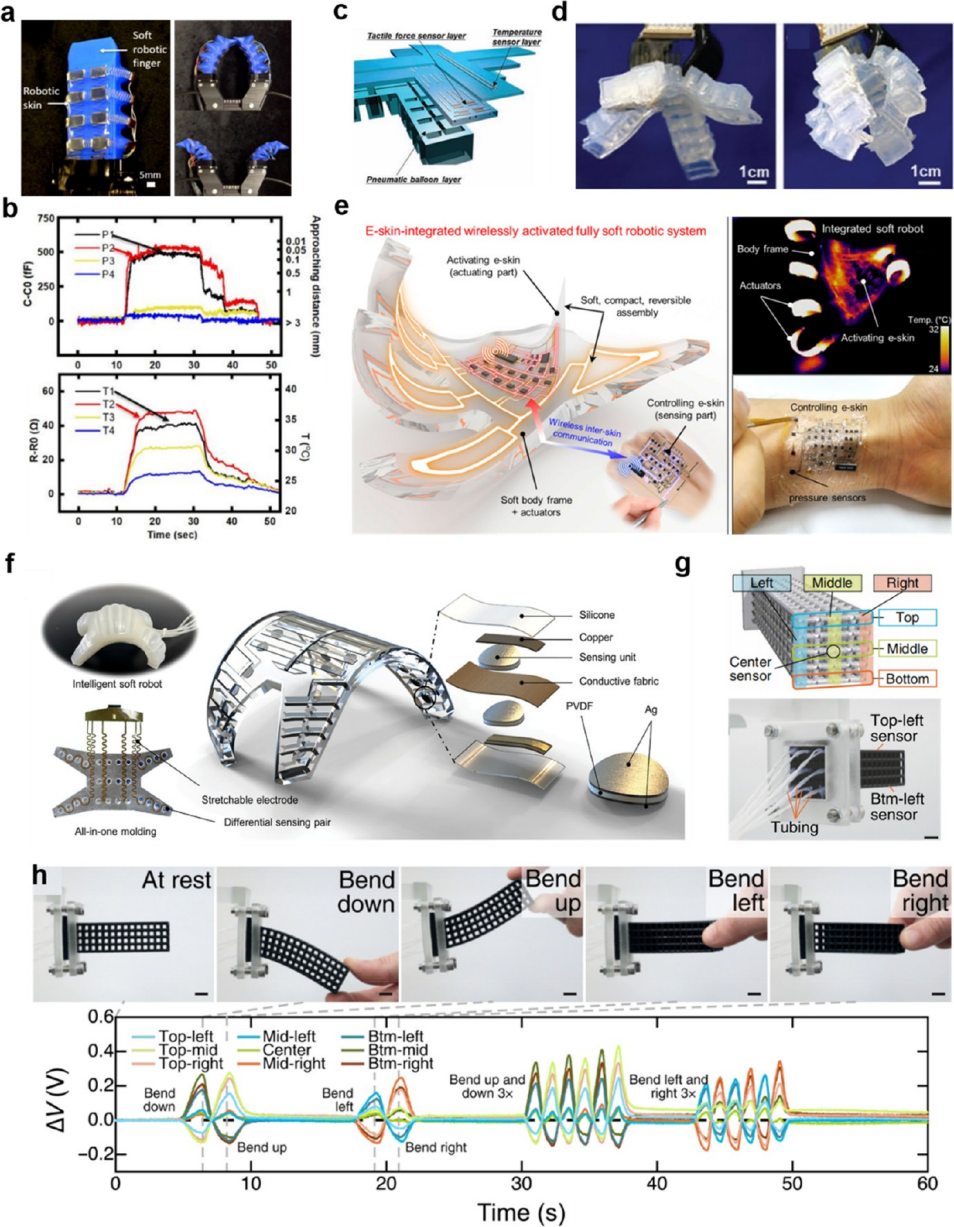
Integrated multimodal sensors on soft robots. (a) Left: Image of a pneumatic soft gripper integrated with a proximity and temperature sensor network. Right: Image of a multisensory gripper in closed and opened states. (b) Plots of corresponding proximity and temperature signals when the gripper touches the doll. Panels a and b reproduced with permission from ref (139). Copyright 2022 The Authors under Creative Commons Attribution 4.0 International License, published by Springer Nature. (c) Schematic illustration of the soft robotic hand integrated with tactile force sensor layer and temperature sensor layer. (d) Image of the skin-integrated soft robotic hand in opened (left) and closed (right) states. Panels c and d reproduced with permission from ref (140). Copyright 2019 The Authors under Creative Commons Attribution 4.0 License, published by Wiley-VCH Verlag GmbH & Co. KgaA, Weinheim. (e) Left: Schematic illustration of fully soft robots mediated by e-skin. Right top: Thermographic image of the activated soft robot. Right bottom: Image of the e-skin interacting with human for user-interactive pressure mapping. Reproduced with permission from ref (141). Copyright 2018 American Association for the Advancement of Science. (f) Illustration of shape-sensing electronic skin for soft robotic proprioception and exteroception. Reproduced with permission from ref (142). Copyright 2023 Wiley-VCH GmbH. (g) Top: Schematic illustration of the distribution of sensors in the soft robot. Bottom: Image of the sensorized lattice from behind with tubing running from each sensor. (h) Top: Photograph of the sensorized soft robot with different bending states. Bottom: Corresponding voltage change as a function of time. Panels g and h reproduced with permission from ref (143). Copyright 2022 The Authors under Creative Commons Attribution 4.0 International License, published by American Association for the Advancement of Science.

Ham et al.^139^ reported a multisensory pneumatic soft gripper integrated with a proximity and temperature network, as shown in Figure 8a. This multimodal sensor network was fabricated on a flexible metalized film, and the electrodes were designed in a Kirigami fashion to improve their stretchability. This multisensory pneumatic soft gripper was demonstrated by touching a doll at different temperatures. The temperature and proximity information when touching the doll can be measured (Figure 8b) wherein combination of both sensors is useful for human–robot interaction. As illustrated in Figure 8c,d, the human-like electronic skin-integrated soft robotic hand proposed by Yamaguchi et al.^140^ was composed of three layers: a pneumatic balloon layer, tactile force sensor layer, and temperature sensor layer. There are 2 × 2 pixels of force sensors that are insensitive to bending on the tactile force layer to measure the object contact and sliding and slipping movements. When the skin-integrated soft robotic hand is actuated to grasp objects, both tactile and temperature information can be detected accordingly for multiple sensing. Despite the intrinsic softness of the soft robotic body, hard modules in the soft robotic systems are common such as circuit boards and pressure-regulating modules.

This hinders the development of soft machines. To solve this problem, Byun et al.^141^ demonstrated fully soft and wirelessly activated robots, whose driving parts, as well as circuits, can be softly, compactly, and reversibly assembled (Figure 8e). Recently, Shu et al. reported a shape-sensing electronic skin^142^ that can measure surface conformations with minimal interference from pressing, stretching, or other stimuli from the environment (Figure 8f). The robot’s movement was tracked from the sensor signal, which was used for 3D shape reconstruction of the soft robot for proprioception. Moreover, machine learning was employed by the robot to recognize different terrains. This is promising for the exteroception of soft robots for more advanced and real-world applications. Recently, Cai et al.^154^ reported a multifunctional e-skin that can measure tactile sensing of pressure and temperature simultaneously with a wide linear response range, which greatly simplifies the signal processing. Moreover, by adopting microprotrusion on a soft substrate, decoupling of these two tactile signals was achieved. As was demonstrated, objects with different hardness and temperature can be recognized by the e-skin-integrated soft robots, attributed to the excellent linear and decoupling characteristics of the sensor. Theoretically, because soft robots have an infinite degree of freedom, it is important to detect multiple deformation modalities of a soft robotic system. By employing a DLP-based printing method, Truby et al.^143^ created sensorized, architected materials and innervating vascular networks for fluidic sensing. The nine fluidic innervation sensing channels in the soft robot can measure a variety of deformations of the soft robot, ranging from bending and stretching to compression, as shown in Figure 8g. Benefiting from the geometrically distributed sensors in the actuator, its bending state can be recognized accordingly. As illustrated in Figure 8h, when the soft robot undergoes different bending states such as bending up, down, left, and right, the relative voltage change for the sensors exhibits a differentiable trend. This provides a possible solution for the proprioception of soft robotics.

**Figure 9:**
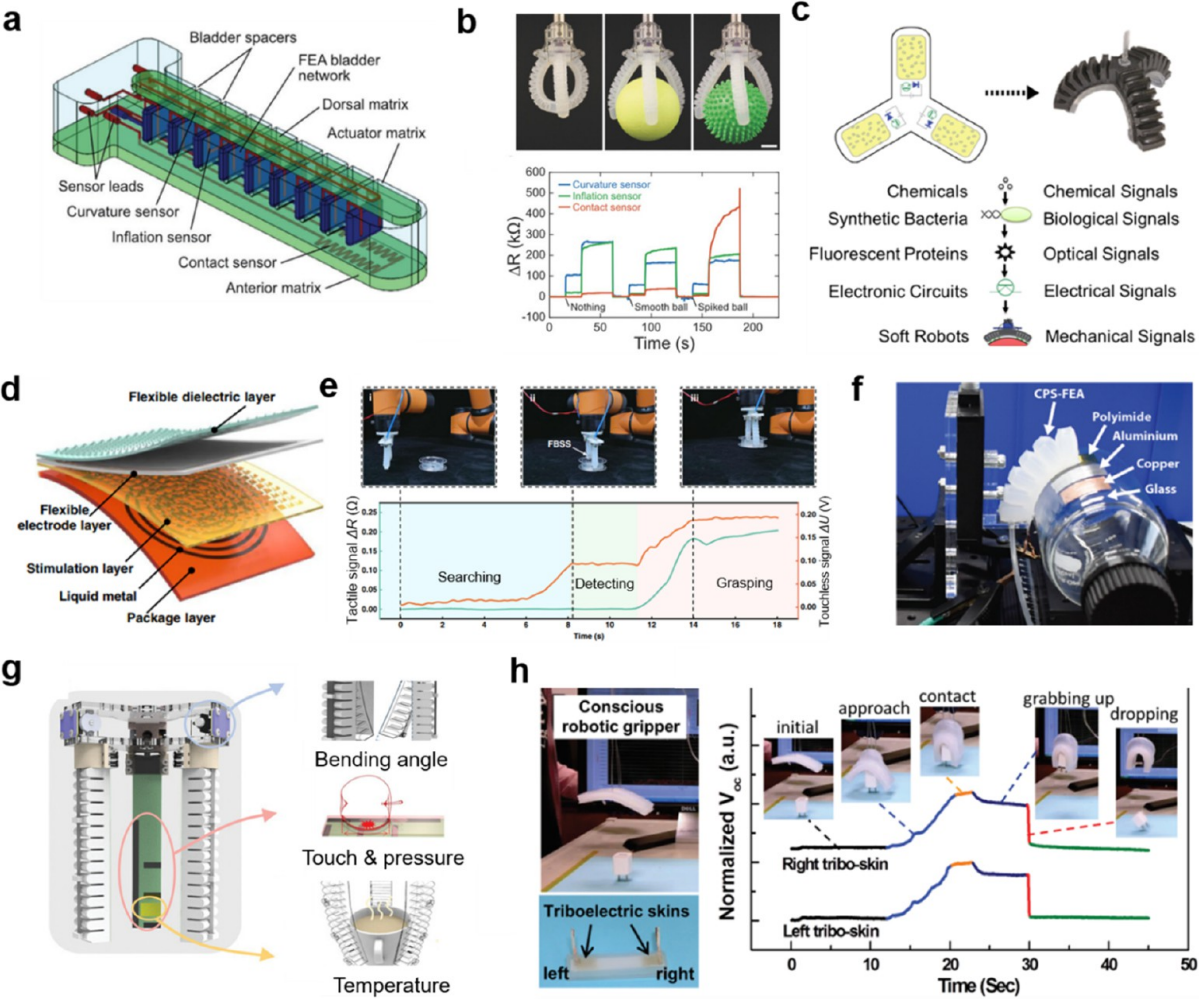
Integrated multimodal sensors on soft robots. (a) Schematic illustration of soft somatosensitive actuator which mainly consists of an actuator matrix and a variety of sensors. (b) Images of the soft somatosensitive gripper holding nothing (top left), a smooth ball (top middle), and a spiked ball (top right) and the corresponding resistance variation from three different types of sensors: curvature sensor, inflation sensor, and contact sensor. Panels a and b reproduced with permission from ref (149). Copyright 2018 Wiley -VCH Verlag GmbH & Co. KgaA, Weinheim. (c) Schematic illustration of chemical-responsive synthetic soft grippers with specific signals. Reproduced with permission from ref (8). Copyright 2019 American Association for the Advancement of Science. (d) Schematic illustration of different layers of the flexible bimodal smart skin. (e) Top: Image of soft robotic gripper integrated with the flexible bimodal smart skin searching, detecting, and grasping a plastic cylinder object. Bottom: Corresponding sensory information from the flexible bimodal smart skin in real time. Panels d and e reproduced with permission from ref (150). Copyright 2022 The Authors under Creative Commons Attribution 4.0 International License, published by Springer Nature. (f) Image of experimental setup for materials recognition by the soft gripper integrated with the sensors. Reproduced with permission from from ref (151). Copyright 2020 The Authors under Creative Commons Attribution 4.0 License, published by Wiley-VCH Verlag GmbH & Co. KgaA, Weinheim. (g) Configuration of the sensor-integrated smart manipulator, of which it is composed of three different types of sensors: bending angle sensor, touch and pressure sensor, and temperature sensor. Reproduced with permission from ref (152). Copyright 2021 The Authors under Creative Commons Attribution 4.0 License, published by Wiley-VCH GmbH. (h) Left: Image of conscious soft gripper and triboelectric skins. Right: Real-time outputs of the triboelectric skins when the gripper is grabbing and dropping an object. Reproduced with permission from ref (153). Copyright 2018 Wiley-VCH Verlag GmbH & Co. KgaA, Weinheim.

It should be noted that 3D printing technology is another powerful technology to fabricate soft, perceptive robots. Truby et al.^149^ employed embedded 3D printing technology to fabricate soft somatosensitive actuators, in which a variety of sensors were embodied, such as curvature sensor, inflation sensor, and contact sensor (Figure 9a). When objects with different shapes and features are held, these differences can be measured by the above-mentioned sensors, as shown in Figure 9b. Moreover, they also developed multidegree of freedom soft actuators that have discrete actuation modes by the same printing technology,^155^ which enables a richer working mode of the soft gripper. The proprioception and tactile perception of the printed soft gripper make the closed-loop feedback control of soft robots nearer. Interestingly, a biohybrid soft gripper was synthesized by Justus et al.^8^ by engineered bacteria for chemical sensing in the surroundings (Figure 9c). This can be used for making practical decisions in the pick and place task and also unlocks an opportunity for synthetic biology in soft robotic systems. By using a triboelectric nanogenerator and liquid metal, Liu et al.^150^ developed a flexible bimodal smart skin that exhibits both touchless and tactile sensing (Figure 9d). With the e-skin, the soft robots not only can be taught to perform specific locomotion by human hands but also can achieve search and grasp tasks through tactile and touchless sensing (Figure 9e). Yang et al.^151^ reported low-cost, paper-based electronics for soft actuators. The resistive strain sensors and capacitive proximity sensors were printed on a paper substrate and then integrated into a soft actuator. Except for bending curvature and distance sensing, this actuator was also used for differentiation of four materials successfully: polyimide, glass, aluminum, and copper (Figure 9f). In order to advance robotic-related industrial automation technology, Sun et al.^152^ proposed an embedded multifunctional perception system based on triboelectric nanogenerator for tactile and bending sensing and pyroelectric temperature sensor for temperature sensing, as shown in Figure 9g. With the aid of machine learning, this soft robotic perception system can achieve an object recognition accuracy of 97%. Lai et al.^153^ also developed a triboelectric-based skin that can actively sense proximity, contact, and pressure to external stimuli via self-generating electricity (Figure 9h). Without the need to provide an electricity source, this active sensing capability can be further used for soft robots and other e-skin-related applications.

## Signal Processing and Feedback Control

**Scheme 1:**
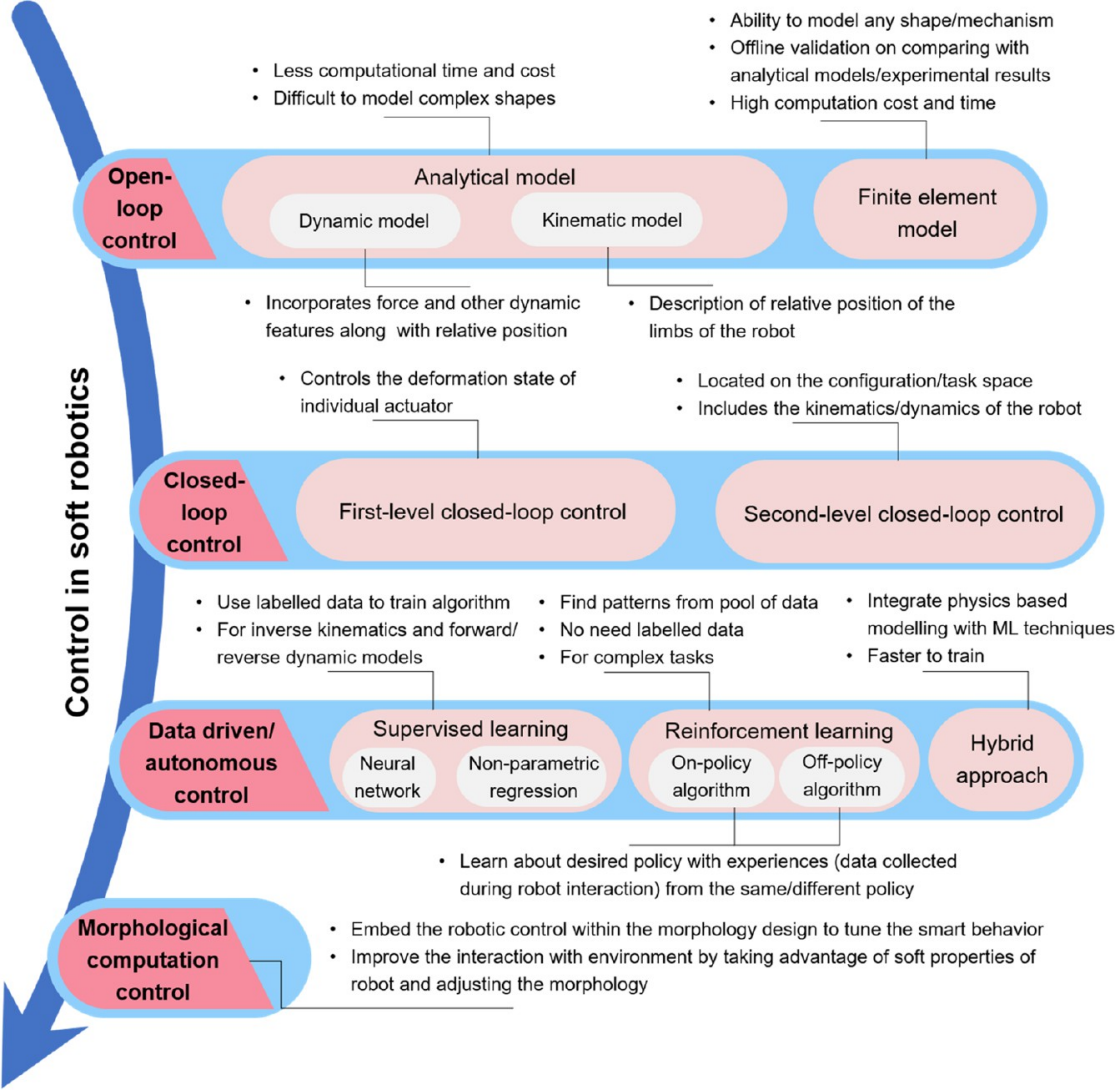
Various Signal Processing and Control Strategies Employed for Soft Robotics Control

Unlike rigid robots, soft robots lack rigid joints, making it difficult to track their motion. Furthermore, their interaction with the environment can lead to deformations, further complicating the task of determining their precise location. However, by integration of sensors into their design, the sensory perception and overall functionality of soft robots can be significantly improved. In this context, signal processing of the sensor data and implementation of algorithms to control the robot are critical to proper functioning of the soft robot. Scheme 1 illustrates various signal processing and control techniques used in soft robotics. A brief description of various modes of robot control commonly employed for soft robot control is provided in the following paragraphs.

### Open-Loop Control

Open-loop control of the robot is achieved by adjusting the controller input without any feedback of the status of the robot. Consequently, this type of control essentially requires extensive knowledge about the shape, dimensions, and material properties of the robot, as well as detailed prior knowledge of the environment in which the robot operates.^156^

In order to describe the state of soft robots, approximation methods are employed due to their potentially infinite degrees of freedom (DOF). Commonly used methods include discrete models, such as the piecewise constant curvature (PCC) or piecewise constant strain (PCS) models. The PCC model divides the robot into small sections with nearly identical curvature, while the PCS model incorporates sections with identical strains. Analytical models are then applied to each section to derive the overall behavior of the robot. In-depth discussion on these topics can be found in a recent article^157^ by Rus’ group. After the state of the robot is characterized, a model can be created using either a kinematic or dynamic approach. The kinematic model focuses on describing the relative positions of the robot’s limbs without considering forces. As a result, this model is relatively simple and can effectively capture the robot’s behavior. Control of the kinematic model^158^ can be achieved through either forward or reverse control. In forward control, input parameters are determined and the corresponding output is obtained based on the model. On the other hand, in reverse control, the desired output is provided to the model, which then calculates the required input using inverse kinematic relations. This input is then used to actuate the controller. Dynamic models, in contrast, take into account the effects of forces^19^ such as gravity and external forces on the robot’s status. Consequently, they offer a more accurate representation of the soft robot’s state.

Another approach for modeling soft robots is through the use of finite element modeling (FEM) to predict their motion behavior. FEM offers the advantage of easily accommodating complex-shaped robots with a wide range of material properties. However, this technique comes with high computational cost and time requirements. Often, FEM models are verified offline in conjunction with analytical models or experimental observations.

In recent years, there has been growing interest in employing fractional order controllers for soft robots. Traditional approaches rely on rigorous models to describe the complex dynamics of the robots. However, these models may overlook certain nonlinear behaviors exhibited by the robots under specific circumstances. Fractional calculus, which allows for the use of real numbers as exponents (instead of just integers), has been utilized to design more robust controllers in engineering applications. Monje et al. provides an in-depth discussion and exciting possibilities of this domain in their recent review.^159^ In summary, open-loop control relies on analytical/finite element models to achieve the desired control.

### Closed-Loop Control

This type of robotic control extensively uses the feedback from the sensors to accomplish accurate control of the soft robot. In this approach, the same models used in open-loop control can be utilized but with the additional integration of sensor data. Unlike open-loop control, where the controller has no means of confirming the desired actuation, closed-loop control incorporates sensor data to determine the success, failure, or partial implementation of the desired actuation. Sensors in closed-loop control measure two types of parameters. First, they sense the input variables of actuation itself, such as voltage, pneumatic pressure, or temperature. Second, they measure output variables such as position, bending angle, temperature, chemicals, proximity, and exerted forces. The signal processing and control algorithm in closed-loop control include an additional set of instructions to adjust the input variables based on the sensor data. In contrast to open-loop control, where any degradation in robot performance cannot be accounted for due to the lack of active monitoring of output variables, closed-loop control allows for the incorporation of advanced algorithms within the control system. These algorithms leverage real-time monitoring of the sensor data, enabling the manifestation of desired functionalities and addressing any potential performance issues in real time.

**Figure 10:**
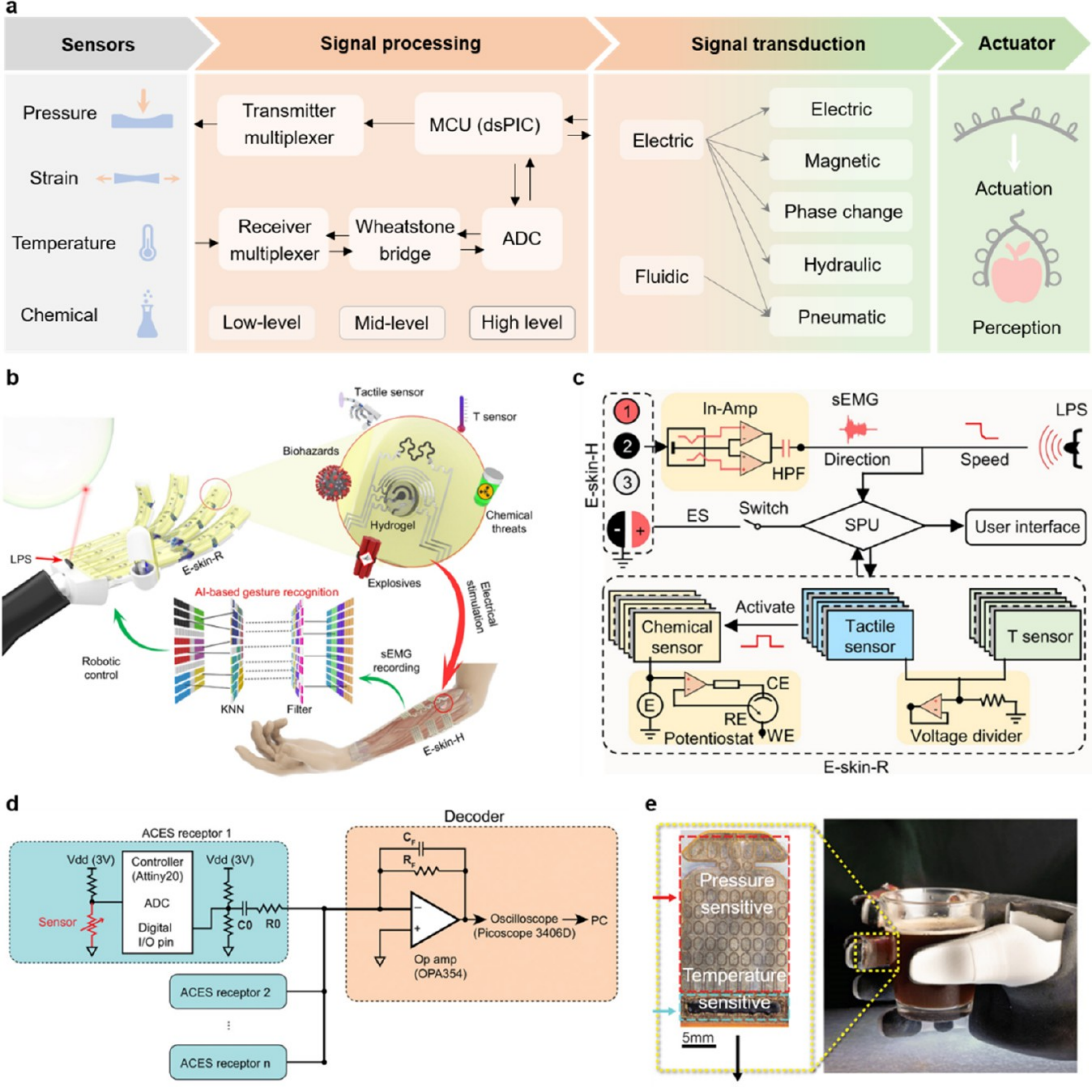
Some relevant signal processing techniques in soft robotics. (a) Workflow of signal processing and subsequent actuator control based on sensor data. (b) Integration of soft e-skin on a robotic fingers and hand for multimodal detection of touch, proximity, temperature, and hazardous substances (c) and the corresponding electronic circuitry for data processing of the collected data from sensors. Panels b and c reproduced with permission from ref (160). Copyright 2022 American Association for the Advancement of Science. (d) Electronic circuitry for data processing of multiarray sensor of asynchronously coded electronic skin (ACES) and (e) corresponding integration of the pressure and temperature sensor array on a robotic hand. Panels d and e reproduced with permission from ref (148). Copyright 2019 American Association for the Advancement of Science.

The typical architecture of signal processing in soft robotics is illustrated in Figure 10a. A myriad of sensory information (such as pressure, strain, temperature, and chemical signal) from various sensors is collected with the receiver multiplexer. The analog signals from sensors are converted into digital format for processing by an analog-to-digital converter (ADC), which further enables subsequent digital signal processing techniques to be applied. To bridge the gap between the raw sensory information and corresponding abstractions, several tools are usually involved in this process, such as proper algorithms, information theory, and machine learning. Specifically, algorithms can be used to extract useful information from a pool of data, and machine learning plays an essential role for making sense of these data. Together they work synergistically to bring soft robotics sensing capabilities to human-like performance levels. It should be noted that there is an interface between the controller (for signal processing as mentioned above) and the actuator. Benefiting from the fast advancements of a variety of actuation mechanisms, such as magnetic, phase change, and electric, more and more opportunities could be provided for the controller–actuator interfacing of soft robotics.

One such implementation of sophisticated signal processing for a closed feedback loop is demonstrated by Yu et al. in their e-skin^160^ capable of physicochemical sensing. The smart robot, as depicted in Figure 10b, is equipped with two conformal e-skin patches: e-skin-R on the robot’s fingers and e-skin-H on its hand. These e-skins were inkjet-printed by using custom formulations. The electronic circuitries of both e-skin-R and e-skin-H are illustrated in Figure 10c. For e-skin-H, surface electromyography (sEMG) data were acquired from four channels using an open-source hardware shield. The sampled signals, ranging from 0 to 1023, obtained from a 10-bit analogue-to-digital converter (ADC), were processed through a serial port. Signal processing is performed asynchronously to the signal acquisition, involving a high-pass filter with a cutoff frequency of 100 Hz and subsequent downsizing using an RMS filter. The resulting peaks from this processing stage were utilized in a machine learning model, which was evaluated for accuracy by using freshly generated data from different gestures. The laser proximity sensor was operated by using custom-built Python software. Furthermore, e-skin-R demonstrated the capability of tactile, proximity, temperature, and electrochemical sensing of explosives, pesticides, and biohazards. Upon signal detection by e-skin-R, real-time haptic feedback and threat alarm communication are achieved through the electrical simulation of the human body using e-skin-H. In another interesting work, Lee et al.^148^ demonstrated ansynchronous coded electronic skin (ACES) which enabled simultaneous transmission of thermotactile information while maintaining exceptionally low readout latencies, despite having an array size of more than 10000. The electronic circuitry for their ACES signal processing is shown in Figure 10d. Conventionally, the sensor arrays are interfaced via time-divisional multiple access (TMDA) where the data collection is performed sequentially and periodically even in the absence of an event. However, in this report the authors use event-based signaling to collect sensor data akin to biological mechanoreceptors. This enhanced their usage of the available bandwidth for signal communication, resulting in successful transmission of the sensor signal asynchronously at a constant latency of 1 ms, with an ultrahigh temporal precision of <60 ns, enabling rapid tactile perception. The working mechanism of the ACES is as follows^148^ “Each receptor in the system comprises a resistive sensor, a microcontroller, and various passive components responsible for signal conditioning. A potential divider circuit converted the resistance to voltages which are sampled by an analog to digital converter. The sampled values are subsequently passed on to firmware models designed to replicate the adaptation behavior of receptors found in human skin, specifically the fast (FA) or slow (SA) adaptation. The ACES-FA model operates by generating an event whenever the measured voltage has changed by >50 mV since the last transmitted event. Following the transmission of an event, a new voltage baseline is established. To mimic SA mechanoreceptors, the model generates events at intervals proportional to the 8-point averaged ADC value. The pulse signature is generated by toggling a digital pin at specific time intervals. To ensure that only the high-frequency components of the signal are transmitted, a capacitive high-pass filter is utilized, resulting in the transmission of voltage pulses. The pulses from multiple receptors are combined by using an inverted summing circuit constructed with an OPA354 operational amplifier. The resulting signal is then digitized at a resolution of 8 bits and a sampling rate of 125 MHz using an oscilloscope. The decoding process is performed offline, utilizing MATLAB.”

In a study conducted by Sonar et al., closed-loop feedback control^161^ of a self-sensing soft pneumatic actuator with soft strain sensors was achieved. The actuator was capable of operating at frequencies up to 100 Hz and generating output forces of up to 1 N. To regulate the input pressure and inflation amplitude, a PID controller was employed. In another investigation conducted by Gerboni et al., a commercial flex bend sensor^162^ was utilized for closed-loop control of flexible fluid actuators. The flex bend sensor detected the curvature, which was then processed by an onboard microcontroller and transmitted to the central control system. The control system consisted of primary and secondary controllers. The secondary controllers were integrated into each module to control valve operations, read sensor signals, and facilitate wireless communication. On the other hand, the primary controller, implemented on the MATLAB platform running on a PC, computed the actuator pressure and selected the appropriate valve for pressure regulation. By utilizing a proportional–integral (PI) controller and a low-pass (LP) filter, the researchers ensured null position error under steady-state conditions and achieved dynamic control of the actuator.

**Figure 11:**
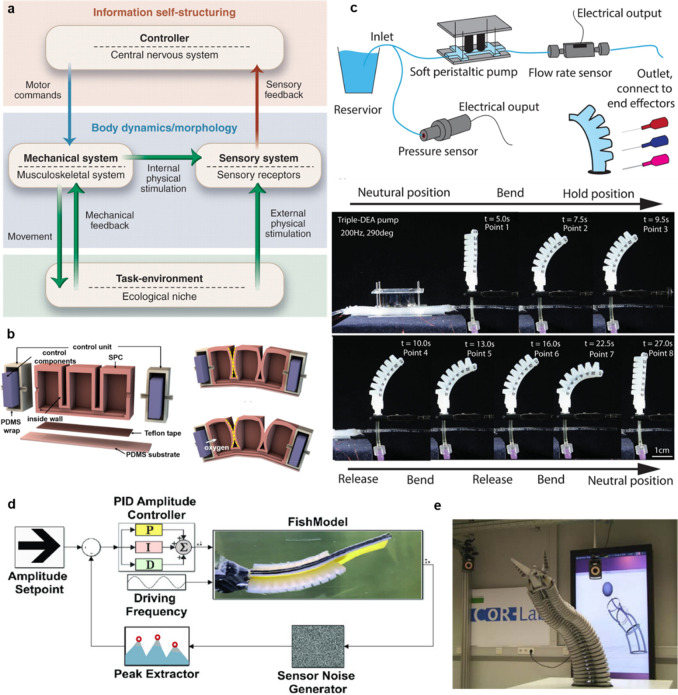
Examples of closed-loop control in soft robotics. (a) Illustration of parallels between a biological system and a soft robotic system in learning. Reproduced with permission from ref (163). Copyright 2007 American Association for the Advancement of Science. (b) Schematic of the soft chemical machine (SCM) showing various components such as pneumatic chambers, control components, fuel, and deflation chambers. Reproduced with permission from ref (164). Copyright 2019 Elsevier Ltd. (c) Top: Circuit diagram of the peristaltic pump equipped with pressure and flow sensor for closed-loop control of a soft robotic finger. Bottom: Demonstration of actuation of soft robotic finger operated by centimeter scale peristaltic pumps. Reproduced with permission from ref (165). Copyright 2023 American Association for the Advancement of Science. (d) Model flowchart for closed-loop control of soft robotic fish. PID controller is used to regulate pressure based on sensor data. Reproduced with permission from ref^166^. Copyright 2023 The Authors under Creative Commons Attribution 4.0 License, published by Wiley-VCH GmbH. (e) Bionic handling assistant (soft robotic trunk) from Festo. The expansion of air chambers is monitored by cables running along the outer hull of each chamber. Reproduced with permission from ref (167). Copyright 2016 Elsevier.

The importance of training and learning in the real world^163^ with the embedded sensor is rightly argued by Pfeifer et al. in their review. While programming a robot to function within a known environment and perform desired motions may seem straightforward, challenges arise when the terrain becomes complex and the external stimuli are unknown. Under such circumstances, extensive training becomes imperative for the robot to function effectively. This is similar to biological systems, as shown in Figure 11a. In a biological system, locomotion learning takes place dynamically over many years. The central nervous system maintains close communication with the musculoskeletal system and sensory receptors to explore and interact with the real world. The interaction between the biological system and its surroundings, encompassing factors such as terrain, temperature, weight, roughness, softness, and force, generates pertinent sensory information that enhances the learning process for each action. Likewise, for a soft robot to be versatile, it is crucial to equip it with sensors and subject it to extensive training encompassing a wide range of external stimuli possibilities.

Chen et al. reported an untethered soft chemomechanical robot^164^ that functions as a well-controlled soft chemical machine (SCM). The conversion of chemical to mechanical energy was achieved by MnO_2_ catalyzed decomposition of hydrogen peroxide, which is used for performing soft robotic actuation (Figure 11b) with ∼33% volument expansion. SCM comprises a reaction chamber, fuel chamber, and deflation chamber, strategically designed to regulate the inflation of pneumatic chambers. To achieve untethered robotic actuation and control, the release of fuel (hydrogen peroxide) is precisely managed using an Arduino nanoboard. An algorithm governs the fuel release based on pressure sensors that monitor the pressure within these chambers. In another notable study, Xu et al. achieved closed-loop control of centimeter scale peristaltic pumps^165^ for powering soft robots. They employed a series of high power density dielectric elastomer actuators as soft motors, which, when operated in a programmed fashion, could produce pressure waves. The soft pump demonstrated the capability to reach pressures of 12.5 kPa while maintaining a flow rate of 30 mL/min, with a response time of less than 0.1 s. To enable closed-loop control of the pressure and flow rate for soft gripper actuation, the researchers utilized pressure and flow rate sensors (as depicted in Figure 11c) in conjunction with a fluid structure interaction finite model. To showcase the versatility of their model, they tested the performance of the peristaltic pump with six commonly encountered liquids, each with varying viscosities. Lin et al. recently presented an intriguing study that showcased the implementation of feedback control for locomotion in a soft robotic fish^166^ (depicted in Figure 11d), incorporating embedded strain sensors. The robotic fish was equipped with antagonistic fast-Pneunet actuators accompanied by hyperelastic gallium–indium embedded within silicone channels to serve as strain sensors. The use of liquid metal as a strain sensor offers advantages such as minimal signal interference from ambient pressure and reduced susceptibility to temperature fluctuations, unlike traditional strain gauges. To accurately predict the motion of the soft robotic fish, a lumped parameter approach was employed, treating the soft silicone structure as a series of interconnected rigid elements. The model’s predictions were compared to experimental observations during calibration, leading to the introduction of appropriate corrections in the control algorithm. For closed-loop control, the strain sensor data were utilized to regulate the pressure within the actuators using a digital pressure regulator. This real-time pressure regulation was achieved through the implementation of a proportional–integral-derivative (PID) controller, while the overall sensor and feedback control system were managed by an Arduino microcontroller.

### Autonomous Control

In the context of closed-loop control, a predefined model of the robot is utilized to determine control parameters based on the current status of the robot and sensor data during actuation. Autonomous control takes a step further by allowing the model of the robot to undergo amendments, enabling more effective functioning in unknown terrains. This is made possible through advanced learning algorithms that leverage sensor data to understand the surroundings, compare the state of the robot’s actuation to expected values, and make appropriate adjustments to the model. These adjustments are achieved through various analytical optimization techniques and machine learning methods. Learning-based methods have the advantage^168^ of considering the nonlinear behavior of soft robots, which can be challenging in model-based control. However, extensive data collection is essential to train robots using this approach.

There are two distinct ways to accomplish this training. First, qualitative modeling or data-learning-based modeling relies primarily on data generated during exploration. Supervised learning and reinforcement learning techniques are commonly adopted within qualitative modeling. In supervised learning, an algorithm assigns cause and effect parameters and the training data set is used to accurately predict the cause and effect relationship through regression. Supervised learning can be performed using neural networks or nonparametric regression. In contrast, reinforcement learning does not preset cause and effect parameters. Instead, the algorithm itself seeks to map the patterns of cause and effect relationships between parameters during exploration. As a result, reinforcement learning can adapt to learning more complex data while exploring unknown terrains with soft robots. It is important to note that learning-based approaches require an extensive amount of data sets to effectively train the robot, and their performance is reliant on the quality of the trained data. Venturing into completely unknown (untrained) spaces can result in the ineffective functioning of the soft robot. In such cases, a hybrid approach to robotic control proves useful.

In the hybrid approach,^156^ data learning methods are combined with physics-based modeling. Physics-based modeling establishes a cause–effect relationship protocol for the robot, while qualitative learning approaches train the nonlinear behavior of the robot, which may not be captured in the modeling process. In this approach, the learning data set can be directly obtained from the mathematical model itself. For instance, the researchers examined the Bionic Handling Assistant (BHA)^167^ developed by Festo (Figure 11e), aiming to enhance the control accuracy of the system. To achieve this, a hybrid modeling approach was employed, combining both forward and error models. The authors demonstrated that incorporating feed-forward control utilizing the inversion of a hybrid forward model, along with a learned error model, significantly improved the accuracy of the system. The proposed approach was specifically showcased through the inverse kinematic control of a redundant soft robot. A hybrid model was constructed by integrating continuum kinematics with an efficient neural network error model.

### Morphological Computational Control

In contrast to conventional model-based control and machine learning based controls discussed in the previous section, morphological computational control^169^ tries to simiplify the control protocols by exploiting the potential drawbacks of the soft robots, viz., under actuated dynamics, non-linear behavior, having infinite degrees of freedom, and being prone to saturation and drift. This is achieved by careful design of the morphology of the soft robots in such a way that key functionalities of the soft robot can be achieved without separately controlling various parts of a robot. For example, a soft gripper comprising a balloon filled with ground coffee easily transitions between a soft conformal robot to a stiff gripper upon applying vacuum. In this case, the key functionalities are embedded within the design and no separate control is necessary. In another report, Corucci et al. demonstrate the use of morphological control^170^ in controlling the motion behavior of the soft silicone octopus robot for the same digital control signal. By tuning 24 morphological parameters of the robot, such as stiffness, damping coefficient, weight, etc., they were able to extract different types of actuation from the soft robot. In this case the control signal remained the same; however, the different behavior was born off the morphological characteristic of the robot. In a similar fashion Judd et al. showed use of a soft arm of the octopus robot to actively sense^171^ the surroundings. Upon actuation of the arm, the waves created by the arm can reach nearby objects, and the rebound waves created a measurable signal in the bending sensor of the soft robot. In this robot, the sensing was accomplished by active motion of the arm, in contrast to conventional passive sensing. Certainly, this approach to robotic actuation, sensing, and smart integration of actuator, sensor, and their control is interesting, and there is an ample opportunity for innovation in this direction.

## Application of Soft Robotic Grippers with Sensing Capabilities

In the last section, different types of sensing mechanisms used in soft robotic actuators were discussed. Here we discuss a few of the reported applications of smart soft robotic actuators.

**Figure 12:**
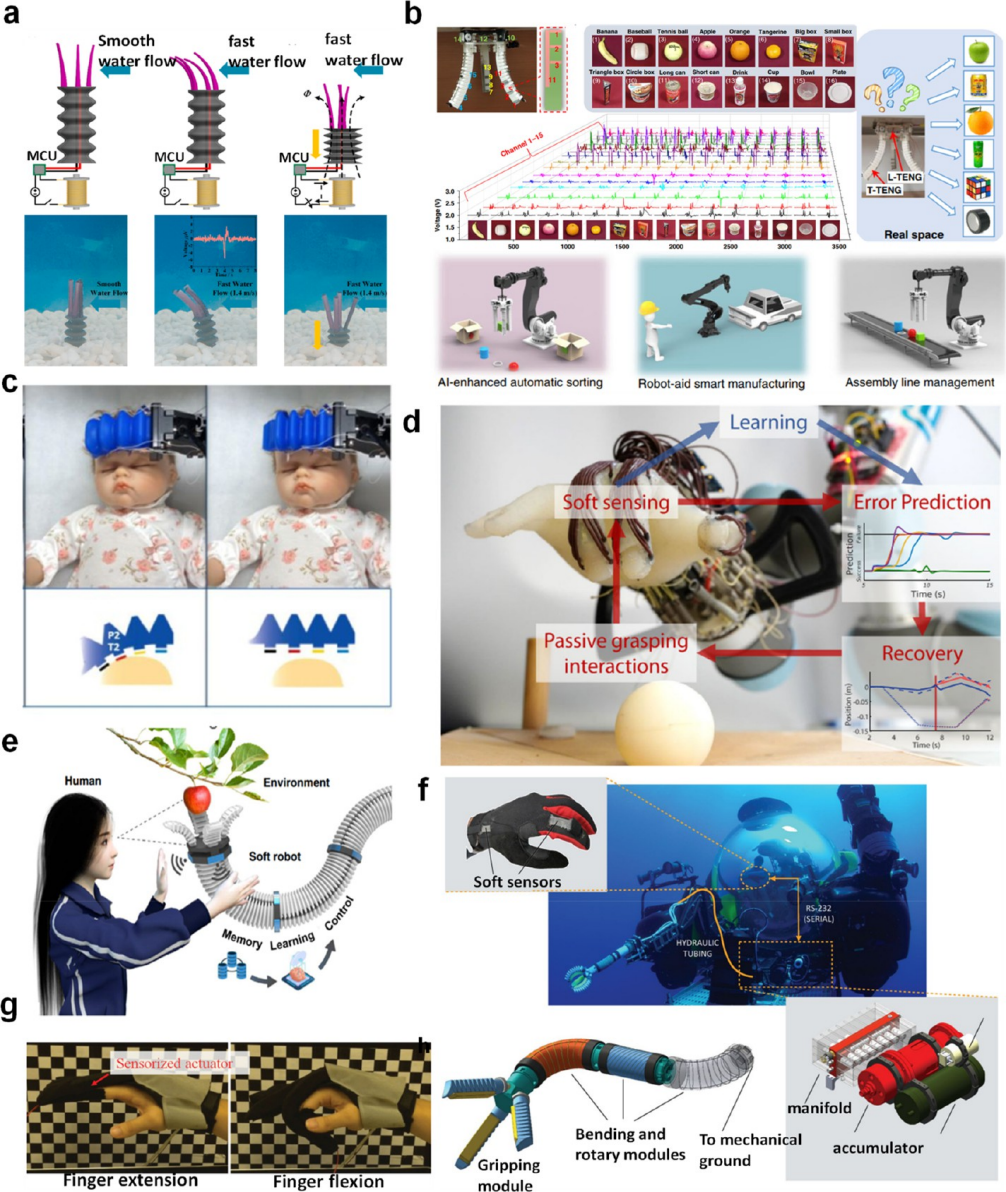
Various applications of soft robots with sensing capability. (a) Sea-anemone-inspired bellows gripper that can measure velocity of water and self-regulate the gripper length for stable anchoring. Reproduced with permission from ref (172). Copyright 2021 Elsevier. (b) Soft gripper equipped with a TENG sensor and running on a machine learning algorithm for smart sorting of objects in the warehouse. Top left: Design of the soft gripper with 15 embedded sensors. Middle: Variation of voltage signal across the 15 channels during grasping of various objects. Bottom: Potential applications of soft robot with TENG sensor. Reproduced with permission from ref (176). Copyright 2020 The Authors under Creative Commons Attribution 4.0 International License, published by Springer Nature. (c) Soft gripper with a heat sensor for health monitoring. Reproduced with permission from ref (139). Copyright 2022 The Authors under Creative Commons Attribution 4.0 International License, published by Springer Nature. (d) Soft robotic hand with embedded tactile sensors for error detection and recovery from passive perception. Temporal data from sparse tactile sensors are used to predict and perform adaptive grasping before the failure. Reproduced with permission from ref (177). Copyright 2023 The Authors under Creative Commons Attribution 4.0 International License, published by Wiley-VCH GmbH. (e) Elephant-trunk-inspired arm with a soft gripper for smart harvesting of fruits. Reproduced with permission from ref (150). Copyright 2022 The Authors under Creative Commons Attribution 4.0 International License, published by Springer Nature. (f) Deep-sea exploration with soft robots. Top left: Gloves fitted with soft sensors for controlling the pressure within the gripper. Middle: Deep-sea exploration robot. Bottom: Design of the soft gripper and bending and rotary modules of the robot. Reproduced with permission from ref (178). Copyright 2018 The Authors under Creative Commons Attribution 4.0 International License, published by Springer Nature. (g) Soft robotic gloves with sensors for rehabilitation of patients with impaired mobility of hand. Reproduced with permission from ref (179). Copyright 2016 Wiley-VCH Verlag GmbH & Co. KgaA, Weinheim.

In one interesting application of a soft actuator, the actuator (Figure 12a) was equipped with sea-anemone-inspired^172^ tentacles to sense the water flow velocity. The soft sensing tentacles of the gripper send the signal upon detection of high water velocity, which triggers the electromagnet. The magnetic field of the EM compresses the bottom part which is made of a composite of polymer and magnetic NdFeB magnetic particles, which stops the soft gripper from being swept away. Such soft grippers can be very instrumental in monitoring and sensing underwater exploration and monitoring. In another application of soft gripper, researchers used the soft gripper with sensors for object recognition.^173^ The sensors can determine the size and shape of the objects and sort and pack them in boxes in a warehouse, supporting extensive automation of the packaging industry. There has been increased interest to application of soft robots with sensors in sorting of trash^174^ and recyclable materials.^175^ In this case the use of artificial intelligence in recognition of the shape, color, and size of the objects is very useful. For instance, Jin et al.^176^ used machine learning to analyze the signal from longitudinal and lateral triboelectric nanogenerator (TENG) sensors (Figure 12b) to predict the shape, size, and softness of objects to sort them in an assembly line. This level of intelligence and control can significantly enhance the productivity of sorting and packaging.

Similarly, another interesting usage of soft gripper equipped with temperature sensor (Figure 12c) is monitoring the temperature^139^ of a patient such as a small children (monitoring whose temperature by thermometer is difficult due to their restless behavior) and accordingly prescribing the treatment in real time. Recently, Gilday et al. demonstrated smart manipulation of the objects by a sensorized soft anthropomorphic hand^177^ using a predictive learning approach. The soft anthropomorphic hand (Figure 12d) is embedded with 32 sensing receptors, the data from which were used to predict failures in advance and adapt the grasping action to double the grasping success rate. The exteroceptive and proprioceptive data of the soft modular sensors was used to predict the real-time failure and success rates of the trials using a long short-term memory (LSTM) network. Their work highlights the potential of fully autonomous grasping through sensor feedback. In another application a soft gripper was used on an automated massage robot^120^ that can perform massages and physiotherapy on a patient with accurate control of pressure on the human tissue. The pressure sensor mounted on the gripper conveys the pressure signal to the control unit, which regulates the pressure during the physiotherapy action for effective therapeutic action. Another increasingly widespread application of soft grippers is in the harvesting of fruits (Figure 12e), vegetables, flowers, and other agricultural produce. The smart sensors in the grippers can sense the ripeness, softness, and maturity of the crop and make a decision concerning the harvest of food items. Also, the embedded sensor ensures gentle handling of the plants and the fruits during harvest, unlike the hard robots. Soft robots are finding increasing use in the field of underwater exploration. In one such application, a soft robot with a flexible arm (Figure 12f) with an attached gripper was used as an underwater robot^178^ for deep-sea biological sampling. The system consisted of gloves fitted with soft sensors, which were used for remotely controlling the robot. On application of pressure on the gloves, the hydraulic pressure of the robotic arm and the gripper can be varied, which helps in locomotion and gripping action. Recently the grippers with sensors have also been used for rehabilitation applications (Figure 12g) for patients with reduced functionality of the hands. In this case, the sensor can accurately monitor the force of extension for smart rehabilitation.

Soft robots also find their applications in other areas such biomedical devices, prosthetics, and wearable robots. As mentioned previously, the field of soft robotics involves the use of flexible materials in the construction of robotic systems. However, in the context of biomedical engineering, it is crucial to prioritize biocompatibility and biomimicry, as highlighted in the review by Cianchetti et al.^180^ The materials employed must demonstrate compatibility with the human body over extended periods to ensure both system functionality and bodily acceptance. Several scenarios warrant consideration: first, for externally worn soft robotic devices used occasionally, allergies and contact reactions must be taken into account. Second, when employing such devices internally, the immune response may lead to rejection of the device. Lastly, for long-term implantation of soft robotic devices, it is imperative to address the possibility of long-term immune responses leading to rejection. Furthermore, the mechanical properties of these materials should closely match those of human tissues. For instance, when soft robots are utilized as prostheses, organs, simulators, or implantable replacements, it is necessary to mimic the mechanical properties and functions of the targeted human tissues.

**Figure 13:**
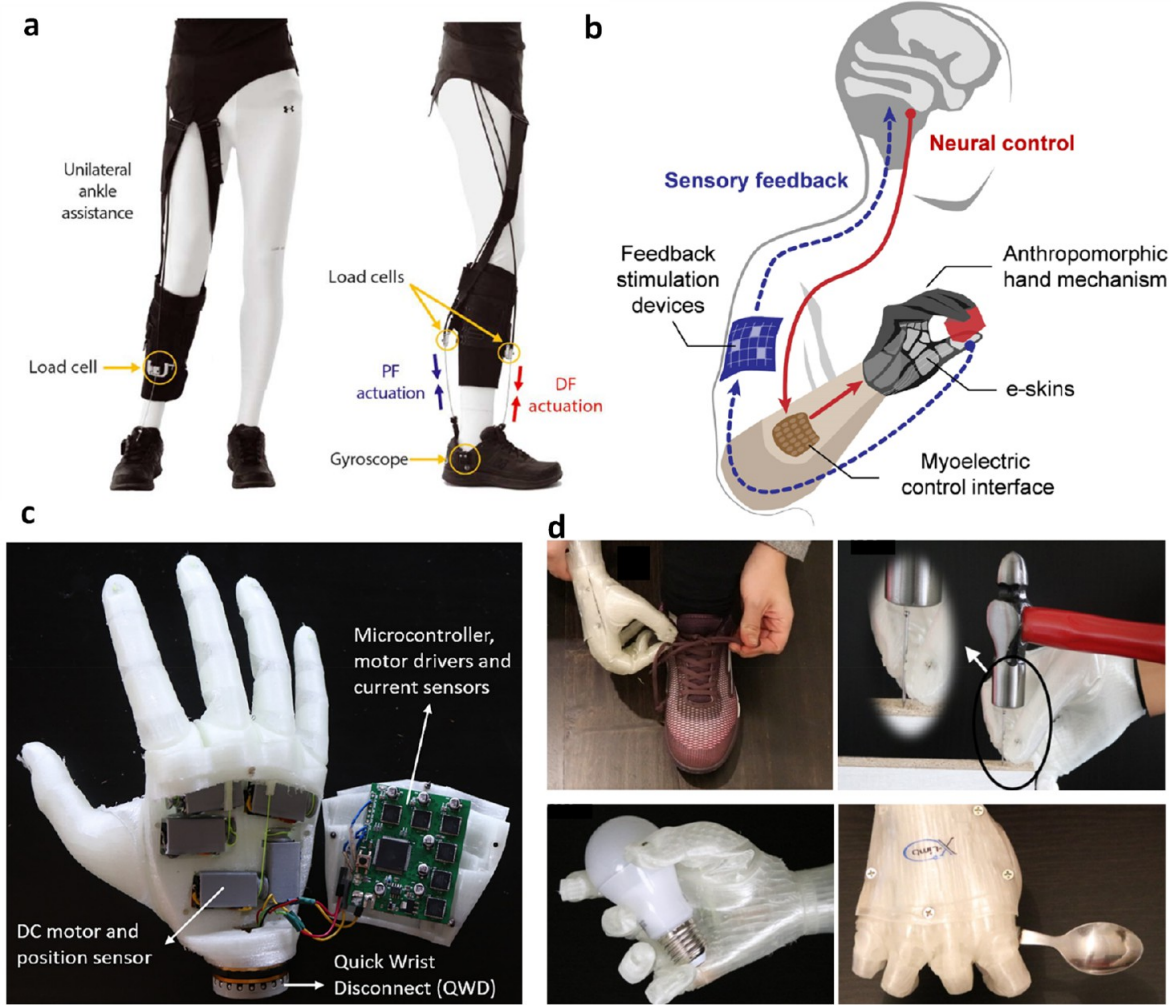
Examples of soft robots with integrated sensors in prosthetics. (a) Soft wearable robot exosuit with attached load cell and gyroscope to augment paretic limb function during hemiparetic walking. Reproduced with permission from ref (181). Copyright 2017 American Association for Advancement in Science. (b) Schematic of integration of neuroprosthetic hand with myoelectric control and sensory feedback interface. Reproduced with permission from ref (182). Copyright 2023 American Chemical Society. (c) 3D-printed soft prosthetic hand with embedded sensors, microcontroller, and drivers. (d) Pictures of soft prosthetic hand being used for a wide range of everyday activities demonstrating its versatile functionality. Panels c and d reproduced from ref (183). Copyright 2020 The Authors under Creative Commons Attribution 4.0 International License, published by PLOS.

One particularly noteworthy application of soft robotics is in the field of wearable assistive and rehabilitation technology. In a study conducted by Asbeck et al. the design and evaluation of a multiarticular soft exosuit^184^ aimed at assisting wearers during walking were presented. The exosuit employed a Bowden cable-driven system connected to an actuation unit to provide the assisting force, while gait events were monitored using a force sensor. Another notable study by Awad et al. introduced a soft robotic exosuit^181^ with integrated sensors (Figure 13a) for timing the gait cycle to aid stroke patients in walking. The external assisting force for the patients was generated through the cable actuation. The sensors, comprising a combination of load cells and gyroscopes, were used to monitor gait events and implemented in a cable position-based force controller, allowing for the modulation of force delivery. These research efforts demonstrate the potential of wearable soft robotics in helping and providing rehabilitation support for individuals in need.

Another significant area of focus in soft robotics is prosthetics. In a perspective article by Gu et al.^182^ the authors emphasized the multidisciplinary challenge of restoring sensorimotor function to upper-limb amputees. Figure 13b shows a schematic of one such approach where myoelectric control of the prosthetic arm can be augmented by use of sensory feedback interface. Providing sensory feedback interfaces is crucial to enable individuals to perceive external stimuli. Various approaches have been reported, including the use of e-skins,^97,185−187^ as well as invasive feedback methods that directly stimulate nerves in the peripheral or central nervous system.^188^ In a study conducted by Abd et al., a prosthetic system^189^ incorporating multichannel feedback signals was developed to convey artificial sensations of touch to the user. The lack of sensory feedback hinders amputees from performing multitasking and fully using the dexterity of their prosthetic hands. To address this, touch sensors, such as BioTac fingertip sensors, were integrated onto the thumb, index finger, and little finger. Arduino microcontroller was employed to process the signals and implement the necessary controllers, including pumps, valves, and pressure sensors. Mohammadi et al. demonstrated the capabilities of a 3D-printed soft robotic^183^ prosthetic hand (Figure 13c) with multiarticulating features (Figure 13d). The hand was fabricated using commercial TPU material through Fused Deposition Modeling (FDM). The finger joints were actuated by DC micromotors connected via cables, and their control was achieved using an open-source Arduino microcontroller. The hand is equipped with two sEMG (surface electromyography) sensors and a position sensor for regulation. The entire prosthetic hand was built at a cost below 200 USD and weighed 253 g. It was capable of generating a power grip of 21.5 N and demonstrated effective performance for over 45000 cycles without any degradation. This design and fabrication approach greatly facilitate access to such advanced prosthetic devices for a wide range of individuals. These advancements in prosthetics aim to provide simulated sensations of touching surfaces, enhancing their overall functionality and user experience. In another report, Zhu et al. demonstrated implementation of in-farbic multimodal actuation and sensing in a soft haptic sleeve.^190^ The sleeve is capable of generating a wide variety of haptic stimuli including compression, vibration, and stretching. The haptic stimuli are generated by controlling the pneumatic pressure inside embroidered stretchable tubes. The grip force is precisely regulated through closed-loop force control using two integrated soft capacitance sensors within compression actuators. Soft robotics has indeed brought about a revolution in the field of surgery, offering numerous benefits, such as increased safety, shorter recovery times, and reduced scarring. One significant advancement facilitated by soft robotics is the widespread adoption of minimally invasive surgery (MIS) as the preferred approach for abdominal procedures.^180^ Tactile sensing plays a crucial role in MIS manipulations, providing essential information to surgeons. In a study by Ozin et al., direct tissue–tool interface sensing^191^ was proven to offer higher accuracy and precision. Sensors placed on the grasping tips enabled the real-time measurement of kinesthetic and tactile forces with improved accuracy. Furthermore, sensors have found utility in medical devices for tissue palpation evaluation. Abiri et al.^192^ conducted a study utilizing a hybrid vibro-tactile feedback sensor to differentiate tissue modulus changes caused by various diseases. By the employment of a multimodal feedback system, a more natural and accurate artificial palpation technique was achieved, facilitating more precise clinical diagnoses. These advancements in sensor technology within soft robotics enable surgeons to have enhanced tactile perception and diagnostic capabilities, thereby improving the overall quality of surgical procedures and patient outcomes.

## Main Industries in the Field of Soft Robotics and Sensing

Robotics has played a critical role in automated manufacturing industry lines, especially in handling heavy and right objects. As a result, these robots are designed to accommodate requirements of maximum load capability, heat resistance, collaborative automation, and precision. Robotics makers such as ABB, KUKA, UR, FANUC, YASKAWA, etc., have been some of household brands. As the usage of robotics moves into the field of soft and delicate objects, there is a need to move into deformable, conformal, and gentle robotics. As reviewed in the previous sections, soft grippers have enabled the handling of soft and delicate objects. However, to give the robots the ability to regulate, sensor feedback is required.

Table 2 presents a few examples of companies that integrate sensors into their robots. The information provided in Table 2 is according to the claims of the respective company in their Web site. We have highlighted them in this work to bring the reader’s attention to few of the leading industries that are active in the field of soft robotics and the extent to which sensor integration have been accomplished. Evidently, a majority of these companies utilize rigid grippers for housing the sensors. These are achieved through coupling a 6-axis sensor for torque and force sensing with the rigid grippers. The rigid grippers possess near-zero force/pressure absorption to translate the change in pressure/force experienced on the end-effector to the 6-axis sensor. In most used cases involving soft and delicate objects, the working force/torque is predefined and feedback controlled by the 6-axis sensors and the electronic controllers. This feedback control gives the rigid gripper the “gentleness” to handle soft objects.

**Table 2:** List of Leading Industries of End-Effector and Sensors in Soft Robotics

Company		Actuation	Gripper Type	Sensing Mechanism	Sensing Params	Multimodality	Sensor—Rigid/Soft Body	Device	Sensor Application
Beijing Soft Robot Tech Co., Ltd. (SRT)^a^	End-effector solution	Pneumatic	Soft	–	–	–	–	–	–
Raruk automation UK^b^		Electrical	Rigid	(1) Torque	mN–N	(6-axis)	Rigid body	Electronics hardware	Feedback control
				(2) Force					
Onrobot^c^		Electrical	Rigid	(1) Torque	mN–N	(6-axis)	Rigid body	Electronics hardware	Feedback control
				(2) Force					
Applied_Robotics^d^		Electrical	Soft	Force	–	–	–	Electronics hardware	Force control
Robotiq^e^		Electrical	Rigid	(1) Torque	mN–N	(6-axis)	Rigid body	Electronics hardware	Feedback control
				(2) Force					
Grabit^f^		Electroadhesion	Soft	–	–	–	–	–	–
Omnigrasp^g^		Electroadhesion	Soft	–	–	–	–	–	–
piSOFTGRIP vacuum-driven soft gripper^h^		Vacuum	Soft	-	-	-	-	-	-
Festo Bionic soft hand^i^		Pneumatic	Soft	(1) Tactile force sensor	–	–	Embedded	Electronics hardware	Feedback control
				(2) Inertial sensor					
Zimmer Group^j^		Pneumatic	Rigid	Finger displacement sensor	0.05 mm	–	–	–	–
Righthand robotics^k^		Electrical	Rigid	–	–	–	–	–	–
Soft Robotics Inc.^l^		Pneumatic	Soft	–	–	–	–	–	–
Rochu Soft gripper^m^		Pneumatic	Soft	Optics	Infrared	–	Mounted	Electronics hardware	Feedback control
Qbrobots^n^		Tendon-driven	Rigid contact, flexible joint	–	–	–	–	–	–
Aidin robotics^o^		Electrical	Rigid	(1) Torque	mN–N	(6-axis)	Rigid body	Electronics hardware	Feedback control
				(2) Force					
SynTouch LLC^p^	Sensors	–	–	(1) Force	–	(1) Force	Mounted soft	Electronics hardware	Feedback control
				(2) Vibration		(2) Roughness			
				(3) Heat flow		(3) Temperature			
Gelsight^q^		–	–	Optics	–	3D surface contour	Mounted soft	Electronics hardware	Feedback control

a Link to company Web site: https://www.softrobottech.com/web/en/category/13.

b Link to company Web site: https://www.rarukautomation.com/collaborative-robots/force-torque-sensor.

c Link to company Web site: https://onrobot.com/en/products/hex-6-axis-force-torque-sensor.

d Link to company Web site: https://www.appliedrobotics.com/products/automation/flexible-smart-gripper/.

e Link to company Web site: https://robotiq.com/products/ft-300-force-torque-sensor.

f Link to company Web site: https://grabitinc.com/.

g Link to company Web site: https://omnigrasp.com/.

h Link to company Web site: https://www.piab.com/en-us/suction-cups-and-soft-grippers/soft-grippers/.

i Link to company Web site: https://www.festo.com/us/en/e/about-festo/research-and-development/bionic-learning-network/highlights-from-2015-to-2017/bionicsofthand-id_68106/.

j Link to company Web site: https://www.zimmer-group.com/en-us/technologies-components/robotics#tab85956.

k Link to company Web site: https://righthandrobotics.com/.

l Link to company Web site: https://www.softroboticsinc.com/.

m Link to company Web site: https://www.softroboticgripper.com/finger-suit/rochu-finger-module-fm-v1.

n Link to company Web site: https://qbrobotics.com/.

o Link to company Web site: https://www.aidinrobotics.co.kr/ultra-thin-torque-sensor.

p Link to company Web site: https://syntouchinc.com/technology/.

q Link to company Web site: https://www.gelsight.com/

However, to handle brittle and delicate objects handling, a soft interface that can conform and deform is required. Festo for example presented a bionic soft hand with bellow-type pneumatic actuation. It has multiple types of sensors integrated: (i) tactile force sensor on the fingers allows real-time force detection and (ii) inertial sensor that allows the measurement of acceleration, inclination, and even vibration. Applied Robotics’ flexible smart gripper incorporates sensors within the soft gripper for accurate control of force during the gripping action. Other companies such as SRT, Soft Robotics Inc., and Rochu, use grippers without sensors and achieve “gentleness” via presetting the pressure required to handle the delicate object. Generally, the gripper is made from hard silicone that can withstand pressure of up to 120 kPa and assembly of a few grippers to work together can even lift items as high as 10 kg or handle soft items such as cakes.

It is worth highlighting the sensor by Syntouch, BioTac, that has a small device profile suitable to be mounted on the tips of rigid grippers and even soft grippers. Due to the triple sensing module inside, it can detect roughness: force exerted/experienced, and temperature. It is suitable for sorting applications that require regulating force of grasp where usage of vision is not feasible. Lastly, GelSight has developed a sensor based on 3D optic profiling. It is tandem direct optic microscopy in which the object compresses an extremely soft gel; as the gel surface deforms conformally to the object surface, the optics measure the 3D profile of the deformation. Owing to extreme sensitivity, the sensor is able to detect the microscopic fingerprints of a finger. However, these soft sensors are housed in a rigid casing, which may not support seamless integration into soft grippers. In summary, (a) the adoption of sensors in grippers is limited to industries that utilize rigid grippers; (b) even though some industries have developed soft tactile sensors, they are encased in rigid casings, which prevent their seamless integration into soft robots; (c) only a few soft robotic companies, such as Festo and Applied Robotics, have successfully incorporated sensors into their soft grippers. Therefore, there is an increased necessity of transitioning smart soft grippers from academia to industry for effective application of soft grippers.

## Outlook

As described in the previous section, the majority of the soft robotic grippers in industry do not incorporate sensors. The majority of the work on smart soft robotic grippers is still limited to academic research and far off from real product development. While researchers have developed a variety of actuation technologies for soft robots with fast response time, high energy density output, and low cost, the development of proprioception and tactile sensing of soft robotics has comparatively lagging behind. Further, multimodal sensing in soft robotics is evidently expected to show increased demand owing to the wide range of applications in which soft robots could be used. Up to date, the sensor integration inside the soft robotic grippers is primarily accomplished by the assembly of the sensors and soft actuators in multiple steps. The need for numerous steps can potentially cause issues concerning the repeatability and reliability of the sensors. A significant improvement in the performance and cost reduction of the smart soft grippers could be achieved by reducing the number of steps in the fabrication of the soft grippers. This would necessitate using technologies such as 3D printing to fabricate embedded sensors within soft grippers in a single step. However, that would require sophisticated multimaterial additive manufacturing fabrication of actuators, sensors, and conductive electrodes in a single step. This creates research opportunities in developing high-performance materials and process development of multimaterial additive manufacturing.

## Vocabulary

**autonomous control**: control of the soft robot without active human intervention, accomplished by use of advanced algorithms which make a decision based on information on the environment from the sensor data
**ionic polymer–metal composites (IPMCs)**: synthetic composite nanomaterials that deform in response to applied electric field and consist of ionic-polymer-like Nafion sandwiched between thin noble metal electrodes, where the deformation is induced by migration of ions due to applied electric field
**kinematics**: study of motion of objects without considering the forces causing the motion and focus on description of motion of joints in terms of velocity and position with respect to surroundings through space and time
**mechanical compliance**: ability of a system to deform or change shape in response to an applied force
**multimodal sensing**: sensing more than one-type of stimulus by a sensor
**triboelectric nanogenerator**: type of energy harvesting device that converts mechanical energy into electrical energy using the principles of triboelectricity, i.e., the generation of electric charge through the contact and separation of two dissimilar materials
